# Development, Pre-Clinical Safety, and Immune Profile of RENOVAC—A Dimer RBD-Based Anti-Coronavirus Subunit Vaccine

**DOI:** 10.3390/vaccines12121420

**Published:** 2024-12-17

**Authors:** Muzaffar Muminov, Nargiza Tsiferova, Egor Pshenichnov, Khusnora Ermatova, Oksana Charishnikova, Alisher Abdullaev, Yuliya Levitskaya, Dilbar Dalimova, Sandhya MVS, Geetanjali Tomar, Ankush Dewle, Pradhnya Choudhari, Aditi Wangikar, Amol Jadhav, Mrunal Mule, Pralhad Wangikar, Ibrokhim Abdurakhmonov, Shahlo Turdikulova

**Affiliations:** 1 Center for Advanced Technologies, Tashkent 100174, Uzbekistan; 2 Institute of Biophysics and Biochemistry, National University of Uzbekistan, Tashkent 100174, Uzbekistan; 3 PRADO—Preclinical Research and Development Organization, Pvt. Ltd., Pune 410506, India; 4 Department of Biotechnology, Savitribai Phule Pune University, Pune 411007, India; 5 Institute of Applied Biological Research and Development, Pune 411007, India; 6 Center of Genomics and Bioinformatics, Academy of Sciences of Uzbekistan, Tashkent 111215, Uzbekistan

**Keywords:** SARS-CoV-2, RBD, vaccine, antigen, toxicology, immunogenicity

## Abstract

**Background:** The development of effective and safe vaccines and their timely delivery to the public play a crucial role in preventing and managing infectious diseases. Many vaccines have been produced and distributed globally to prevent COVID-19 infection. However, establishing effective vaccine development platforms and evaluating their safety and immunogenicity remains critical to increasing health security, especially in developing countries. **Objectives:** Therefore, we developed a local subunit vaccine candidate, RENOVAC, and reported its toxicity and immunogenicity profile in animal models. **Methods:** First, the synthetic gene-coding tandem RBD linked with the GS linker was cloned into the expression vector and expressed in CHO cells. The protein was then purified and filter sterilized, and 10 µg/dose and 25 µg/dose formulations were finally examined for the 14-day repeated dose toxicity followed by the immunogenic profile in preclinical studies. **Results:** When administered to Sprague Dawley rats by intramuscular route, the vaccine was well tolerated up to and including the dose of 25 µg/animal, and no toxicologically adverse changes were noted. The observed change in weight of the thymus and spleen might be related to the immunological response to the vaccine. The dimer RBD vaccine demonstrated the ability to generate high levels of specific immunoglobulins (IGs) and neutralization antibodies (NAbs). Finally, changes in the amounts of specific T cells and cytokines after vaccination suggested that the vaccine mainly triggers an immune response by activating CD4+ Th2-cells, which then activate B-cells to provide humoral immunity. **Conclusions:** The study suggests that, based on its reliable immunogenicity and acceptable safety, the vaccine can be further directed for clinical trials.

## 1. Introduction

Since the first recorded COVID-19 infection on the 31 December 2019, more than 774 million cases have been confirmed. The death toll because of the infection reached over 10 million [1]. This pandemic has forced countries to urgently find solutions in health care, science, and innovations to control the disease. The vaccine development capability and practical large-scale vaccination efforts are essential to pandemic control. Several dozen vaccine candidates are available for mass immunization or phase III clinical trials [2,3,4]. Despite using different approaches and platforms, all available vaccine candidates target the SARS-CoV-2 surface protein and its fragments as antigenic molecules; mainly, its ACE-2 receptor-binding domains (RBD) are considered promising candidates [5,6]. For example, the most widely used vaccines produced by Biontech/Pfizer and Moderna relied on mRNA that expresses spike protein when administered into the human body [7,8]. Meanwhile, the ZF-2001 vaccine, which has undergone clinical trials in Uzbekistan and has been used in mass vaccination on a large scale, is based on the RBD antigen of the new coronavirus crown protein [9,10]. Although the ZF-2001 showed a high safety profile and efficacy rate of 84.8% in phase III clinical trials in Uzbekistan [11] and has been available for shorter relatively periods, the country needs vaccine development platforms for timely responses against infectious diseases and future outbreaks.

Additionally, it is a must to assess the preclinical safety of vaccines and gain a deeper comprehension of how the particular vaccine affects the immune system before proceeding to the clinical stage, to avoid any potentially severe adverse effects. Thus, in this work, we evaluated the toxicity and different parameters of the immune response, such as antibody production, cytokine, and cellular immune response, in laboratory animals under GLP settings, besides covering the basic development stage of the vaccine.

Briefly, the target genes encoding the dimeric RBD protein were first designed and codon-optimized accordingly. The synthetic gene was cloned into the pcDNA3.4 plasmid by ligation, followed by sequence confirmation. After the initial expression studies in CHO cells, the expression of the target protein was further scaled up and purified by two steps of liquid chromatography. Then, the purified target antigen was filter-sterilized and aseptically formulated with an alum adjuvant. Two low- and high-dose formulations and a placebo control were prepared. Further, (i) 14-day toxicity and (ii) toxicity with a 28-day recovery study were performed to evaluate the preclinical safety of the vaccine antigen. Finally, different immune response parameters, such as specific antibody production, neutralizing efficiency, and cellular and cytokine profiles, were studied in mice.

## 2. Materials and Methods

### 2.1. Vaccine DevelopmentProcess

#### 2.1.1. Protein Designing, Expression and Purification

The SARS-CoV-2 spike sequence was retrieved from Genebank (accession number: MN9089473). The antigen was designed with a 5xGS linker between tandem repeated RBD encoding sequences (319–541 amino acids). The native signal peptide was changed for the BM40 signal peptide after checking cleavage efficiency and secretion possibility of several common signal sequences with SignalP v5.0 and Cell-PLoc v2.0 tools [12,13], and a 6-histidine tag was added to aid the purification process. Further, the sequence was codon optimized, and the gene was synthesized (GenScript Biotech, Piscataway, NJ, USA) and cloned into CHO expression vector pcDNA3.4 (Thermofisher Scientific, Waltham, MA, USA).

The plasmid was produced in the *E. coli* Top10 strain, and the antigen was expressed in ExpiCHO-S cells according to the manufacturer’s instructions [14]. Primary expression, verifying the protein expression level, stability, optimized transfection, and purification, was carried out in volumes not exceeding 20 mL of the culture medium. For non-clinical studies, expression was scaled in volumes from 500 to 3000 mL.

The supernatant was collected on day 12, and the antigen was first purified by Nickel affinity chromatography using a HisTrap HP 5 mL column (Cytiva, Uppsala, Sweden) to remove the main impurities. The sample was purified by anion exchange with Macro-Prep High Q Mini 5 mL (BioRad, Hercules, CA, USA). The purified target protein was concentrated with 10 k MCWO centrifuge filters, and the buffer composition was exchanged to phosphate buffer saline, pH-7.4. The purity of bulk antigen was analyzed using SDS-PAGE and SEC-HPLC.

#### 2.1.2. Formulation, Filling, and QC Analysis

Alum adjuvant was prepared by dissolving aluminum hydroxide (Merck, Darmstadt, Germany) in filter-sterilized PBS pH-7.4, followed by autoclave sterilization. First, the antigen molecule was cold sterilized with sterile 0.2 µm PES syringe filters (Thermofisher Scientific, Waltham, MA, USA) and mixed aseptically with sterile PBS pH-7.4 and concentrated alum to get the final concentration of (i) 25 µg antigen and (ii) 10 µg/dose with 0.25 mg aluminum per 0.5 mL vaccine (Table 1). Placebo vaccine was prepared without the antigen. The bulk preparations were mixed at 200 rpm at 37 °C for 30 min to allow adsorption of the antigen on the alum. Then, three formulations were aseptically filled into sterile vials, and critical parameters such as sterility and protein content were confirmed accordingly.

### 2.2. Subject Details and Ethical Aspects of the Study

#### 2.2.1. Laboratory Animals

Six- to eight-week-old Sprague Dawley rats and five- to seven-week old Balb/c mice were both received from the National Institute of Biosciences (CPCSEA Reg. no.: 1091/GO/Bt/S/07/CPCSEA) and were in good health, free from viruses, and were solely used for this study. All animals were housed under SPF conditions in PRADO Ltd. animal facilities and allowed free access to water and a standard rodent diet. Automated systems kept room temperature, humidity, and photoperiod within the recommended range. Both rats and mice were acclimated for five days before the trial commenced.

#### 2.2.2. GLP and Ethics Statement

All animal studies were conducted according to international and national laws and guidelines on nonclinical evaluation of vaccines and good laboratory practices. The animal studies carried out at PRADO Ltd. (Pune, India) are certified by the National GLP Compliance Monitoring Authority (NGCMA) (Certificate Number: GLP/C-168/2021), Department of Science & Technology, Govt. of India, for compliance to OECD-GLP. Both toxicity and immunogenicity studies were approved by the Institutional Animal Ethics Committee (IAEC-23-002&IAEC/23-003). All animal results are reported following ARRIVE guidelines [15].

### 2.3. Toxicity Study in Sprague Dawley Rats

The study design comprised three groups of a 14-day toxicity study: placebo control (G1), low dose (G2), high dose (G3), and two groups of 14-day toxicity study with 28-day recovery: placebo control-R (G4) and high dose-R (G5) (Table 2). All the groups contained 12 animals, six rats/sex/group. They were randomly allocated to control and different treatment groups based on their recent body weights to keep the weight variation of animals less than ±20% of the mean body weight for each sex.

Animals in low dose (G2), high dose (G3), and high dose-R (G5) groups were administered 10 µg and 25 µg of the vaccine per animal, respectively, by intramuscular route on Day 1 and Day 8. Animals in the placebo control (G1) and placebo control-R (G4) groups were administered RENOVAC placebo control. We checked mortality and morbidity twice a day after the first dose administration. Clinical signs and local tolerance were examined daily, and feed consumption, body weights, and detailed clinical examinations were measured and performed once a week. The ophthalmoscopic examination was conducted in G1 and G3 during the last week of the treatment. The low-dose group was omitted, as no ophthalmic abnormal signs were observed in either group. Finally, we conducted hematological, chemical, and urine analyses and assessed the gross pathology and histopathological changes in their organs after euthanasia.

### 2.4. Immunogenicity Studies in Mice

#### 2.4.1. Study Design

**Figure 1:**
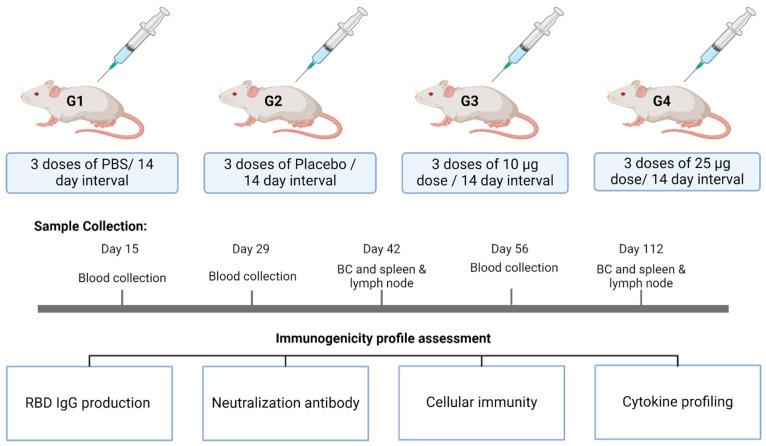
Study design to assess immunogenicity in mice. G1—Vehicle control; G-2—Placebo Control; G3—Low-dose; G4—High-dose. Created in BioRender.com.

To evaluate the immunogenicity of our vaccine, 5–7-week-old male and female Balb/c mice with body weights between 15.5 and 20.5 g were randomly allocated into four different groups: G1—vehicle control (PBS), G2—placebo control, G3—low-dose vaccine treatment, and G4—high-dose vaccine treatment (Figure 1). Each group contained 40 animals, 20 mice/sex/group. The mice were administered three doses of PBS (G1), placebo (G2), 10 µg (G3), and 25 µg (G4) of the RENOVAC vaccine on day 1, followed by two booster doses on days 14 and 28. For the immunization of mice, the intraperitoneal route was chosen over the intramuscular route, as recommended elsewhere [16].

As our goal was to investigate the long-term effect of the vaccine candidate on various immune factors, we conducted the study over a period of 112 days. We collected blood samples from 4 mice per sex, per group, on days 15, 29, 42, 56, and 112 under isoflurane anesthesia. Next, the serum samples were prepared by centrifuging them for 10 min at 2500 RPM. At each time point, anti-RBD IgG levels were measured. However, cytokine profiles and neutralizing antibody titers were checked only after the entire course of immunization on days 42, 56, and 112. The same serum samples were used for three studies and were stored at −80 °C until further analysis. Cellular immunity was evaluated from spleens and lymph nodes collected on days 42, 56, and 112 after terminally sacrificing the animals. The spleens and lymph nodes were processed for sample preparation on the same day of collection.

Other parameters evaluated included mortality, clinical signs, local tolerance, detailed clinical examination, and body weight on days 1, 14, and 28.

#### 2.4.2. Assessment of Anti-RBD IgG Production

For detection of RBD (Anti-SARS-CoV-2) IgG, the GENLISA™ Anti-SARS-CoV-2 IgG Antibody to Spike RBD Protein Quantitative ELISA kit, along with SARS-CoV-2 Spike RBD-His Recombinant protein pre-coated micro-titer 96-well plates, were purchased from Krishgen Biosystems (Mumbai, India). During the measurement, the following dilutions were selected for treatment sera based on the validation study: 1:500, 1:1000, 1:2000, 1:5000, 1:10,000, and 1:20,000 (*v*/*v*), and the final calculations were adjusted to account for the dilution factor. As per the manufacturer’s instruction, 100 µL of standards at different concentrations and diluted sera samples were added to the respective wells, and the plate was incubated for 1 h at room temperature. Following incubation, the wells were washed four times with 1× wash buffer, and then 100 µL of Rabbit Anti-Mouse IgG: HRP-Conjugate was added to each well. The plate was subsequently incubated for 1 h at room temperature (18–25 °C) by sealing it. After incubation, the wells were washed the same way as mentioned previously. 100 µL of 3, 3′, 5, 5′-tetramethylbenzidine (TMB substrate solution) was added to all wells and incubated in the dark for 15 min at room temperature. The reaction was stopped by adding 100 µL of stop solution, and within 30 min, the absorbance rate was measured at 450 nm using an ERBA LISA Scan EM Reader. All the sera samples were processed in duplicate.

#### 2.4.3. Neutralization Antibody Titer Assessment

The GENLISA™ SARS-CoV-2 (COVID-19) Surrogate Virus Neutralization Test (sVNT) ELISA kit was used to quantitatively analyze neutralizing antibodies against SARS-CoV-2 in immunized mice. The sera samples collected on days 42, 56, and 112 were prepared by diluting them with sample diluent at a 1:100 (*v*/*v*) ratio. Initially, 100 µL of negative control, a positive control, standards, and diluted samples were introduced into the blank neutralization reaction plate. Subsequently, 100 µL of the SARS-CoV-2 RBD: HRP conjugate was added to the wells. The plate was then sealed and left at room temperature for an hour for neutralization. 100 µL of each sample solution from the neutralization reaction was transferred into the second human ACE-2-coated microtiter plate. Following a 90-min incubation at room temperature, the wells were washed four times with 1× wash buffer, and any remaining buffer was eliminated by firmly tapping the plate upside down on absorbent paper. Subsequently, 100 µL of TMB substrate solution was added to each well and incubated in the dark for 30 min at ambient temperature. The reaction was stopped by adding 100 µL of stop solution, and the absorbance was measured at 450 nm within 30 min. All samples were processed twice.

#### 2.4.4. Cellular Immunity Assessment in Spleen and Lymph Node

The spleen and lymph nodes were broken up mechanically using frosted slides. The tissues were then passed through a 21-gauge needle to get a single-cell suspension of splenocytes and lymphocytes. The single-cell suspensions were centrifuged at 1200 rpm for 10 min at room temperature. The supernatant was discarded, and the cells were resuspended in PBS, followed by fixation using 3.7% paraformaldehyde (PFA) for 10 min at 4 °C. After washing twice with PBS, the cell suspension was filtered through a cell strainer (40 µm) to eliminate tissue debris and clumps. The cell suspensions were then blocked with 10% FBS for 30 min at 4 °C. After that, antibodies were added at a concentration of 1:100 (1 µL of antibody/s in 100 µL of cell suspension). The mixture was then left at 4 °C overnight. The tubes prepared with about 1 × 106 cells per tube, the anti-mouse CD3, CD4, and CD8 antibodies, as well as the isotype controls for each one and the dilution factors, are shown in Table 3. The samples were analyzed using an AttuneTM NxT Flow Cytometer.

#### 2.4.5. Cytokine Profile Assessment by Multiplexing

The sera samples collected on Days 42, 56, and 112 were subjected to cytokine profiling, including G-MCSF, IFNγ, IL-1β, IL2, IL4, IL5, IL6, IL12p70, IL13, IL18, and TNFα, as per the manufacturer’s protocol using the ProcartaPlex Mouse Th1/Th2 Cytokine Panel 11plex kit. First, 50 µL of vortexed 1× capture bead mix B was added to each well, allowed to settle at the bottom of the plate for 2 min, and washed three times with 150 µL of 1× wash solution. According to the plate layout, equal amounts (25 µL) of UAB solution and undiluted/diluted sera samples were added to the wells. The plate was shortly incubated at RT with shaking at 500 rpm and then stored at 4 °C overnight. The next day, the plate was shaken for 30 min at 500 rpm on the orbital shaker and washed three times with 1× wash buffer. 25 µL of the biotinylated detection antibody mix was added to each well, and the plate was further kept on an orbital shaker for another 30 min. After washing the wells accordingly, 50 µL of Streptavidin-PE (SA-PE) was added to each well and reincubated for 30 min on the orbital shaker. Finally, 120 µL of reading buffer was added after washing, and the plate was read using Bio-Plex 200 Systems with appropriate instrument settings to characterize cytokine profiles.

### 2.5. Data Analysis

All the individual data were summarized in terms of groups and sex to obtain the mean and standard deviation. The main group animals’ body weight, body weight gain, feed intake, urinalysis (pH and specific gravity), hematology, clinical chemistry, and absolute and relative organ weight data were analyzed using a one-way ANOVA followed by Dunnett’s test. The recovery group animals’ data were analyzed using a *t*-test. All the data on vehicle control and placebo control in immunogenicity were analyzed by a *t*-test. Further data from all treated groups was compared with placebo control using a one-way ANOVA. All analyses and comparisons were evaluated at the 5% (*p* ≤ 0.05) level.

## 3. Results and Discussions

### 3.1. Vaccine Development

**Figure 2:**
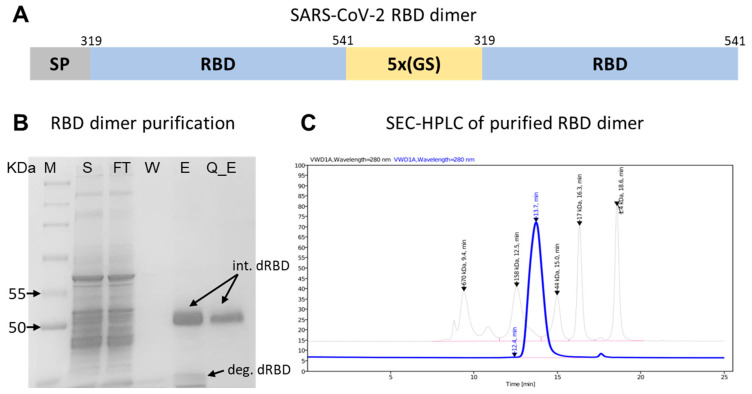
Design of dimer RBD antigen and purification. (**A**) Schematic SARS-CoV-2 dimer RBD antigen diagram. (**B**) Purification of dimer RBD: M—molecular weight markers; S—the initial culture liquid; FT—the protein fraction not bound to the affinity sorbent; W—wash fraction with a buffer containing 40 mM imidazole; E—elution of the target fraction with a buffer containing 250 mM imidazole; Q_E—the elution of the target protein from the ion exchange column. (**C**) SEC-HPLC analysis of purified antigen. Blue line—Dimer RBD; Pale black lines—standard proteins.

Considering the high safety profile of recombinant protein vaccines [17,18,19] and the importance of anti-RBD antibodies in SARS-CoV-2 neutralization [20,21], we aimed to develop a vaccine based on RBD. Previous studies have revealed that the dimer or trimer form has superior immunogenicity over the monomeric form [6,9]. We designed the antigen as an RBD tandem repeat connected with a 5xGS linker (Figure 2A).

Additionally, previous studies and our results have shown relatively efficient production of the receptor-binding domain of the SARS-CoV-2 protein compared to the entire spike protein [22]. The final yield of dimer RBD in our study varied from around 20 mg to 50 mg per liter of culture after the full purification process. In contrast, the spike ectodomain had a much lower yield, which was a limiting factor for further development. However, when expressed in CHO cells, the target dimer RBD protein had an extra band, which we think might be due to the enzymatic breakdown of the dimer into its monomeric form (Figure 2B). The degraded RBD and other minor impurities that existed from the first stage of purification were successfully removed, either by ion exchange due to different binding strengths or by size-exclusion chromatography. The purified target protein exhibited a molecular weight of around 50–55 kDa in gel electrophoresis, and SEC-HPLC verified a purity level of at least 99% (Figure 2C).

### 3.2. Toxicity Study

Mortality, clinical site observation and detailed clinical observation.

Throughout the study, neither mortality nor abnormal clinical symptoms were detected in any of the rats. Comprehensive clinical evaluations suggested the absence of any treatment-related clinical abnormalities in any animals.

#### 3.2.1. Body Weights and Feed Consumption

**Figure 3:**
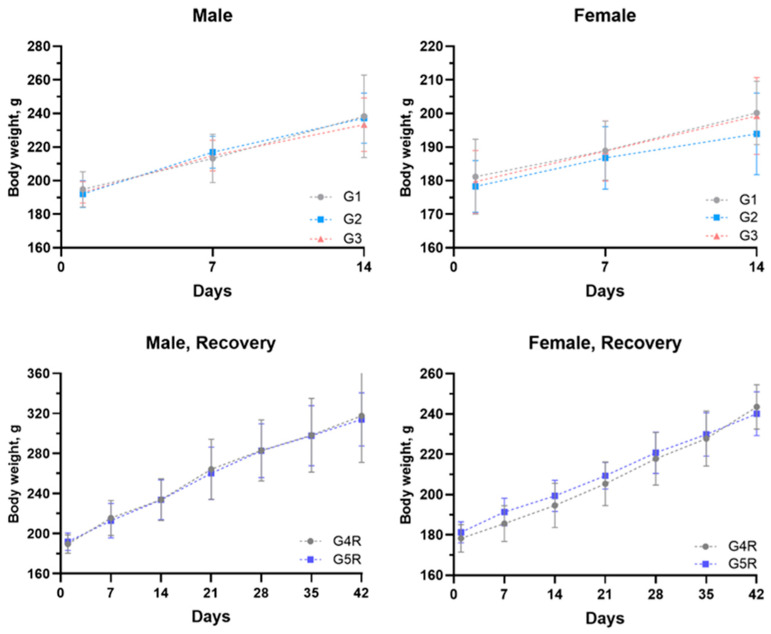
Body weight transformation in rats. The values represent the mean ± SD of all animals per group.

All control and treatment groups exhibited a typical and expected rise in body weight for all animals (Figure 3).

Dose-dependent decreases in body weights on day 14 for the first three groups, and on day 42 in the recovery groups were not statistically significant. Body weight gain did not show consistent patterns across all time points for both sexes (Supplementary Data S1). Hence, it is not considered to be related to the treatment of the test item. Feed consumption in males and females was comparable to that of the control group without any statistically significant changes (Supplementary Data S2).

#### 3.2.2. Hematology

Hematology analysis in males revealed that WBC values were dose-dependently increased on day 15; however, on day 43, they were slightly decreased (Table 4). In the case of females, the WBC values were somewhat improved on day 43 only. There was a statistically significant increase in MCH values only in G2 females on day 15. The minimal increase in HGB and HCT values and decrease in PLT values in the main group females were not considered to be related to the treatment of the test item.

All other hematological parameters (on days 15 and 43) in males and females were comparable with those of controls.

A differential leukocyte count on day 15 revealed a dose-dependent statistically significant increase in neutrophil count and a relative decrease in lymphocyte count in G3 group males (Table 5). The differential leukocyte count in the main recovery group females and recovery group males was comparable with that of respective controls. The changes observed in values of WBC and differential leukocyte count suggest significant immunological activation induced by the vaccine, though not toxicologically adverse. The increase of neutrophils, coupled with the reduction of lymphocytes, likely represents an initial immune response to the vaccine. Neutrophils are often the first responders to infection or immunological stimuli, providing an immediate defense mechanism. In contrast, lymphocytes, particularly in a decreased state, may indicate a temporary redistribution to areas of active immune response or reflect the regulatory adjustments occurring post-vaccination.

#### 3.2.3. Clinical Chemistry

After vaccination, clinical chemistry analysis on day 15 showed a non-significant, dose-dependent increase in glucose (GLU) levels in both males and females (Table 6). Significant increases in alkaline phosphatase (ALP) in males and glutamate oxaloacetate transaminase (GOT) in females were observed, indicating possible mild transient liver stress. Decreases in blood urea nitrogen (BUL, CBUN) in males suggest altered protein metabolism or mild renal adaptations, while a reduction in cholesterol (CHOLE) in females may indicate lipid metabolism changes due to immune activation.

After the recovery period, males showed decreased glutamate pyruvate transaminase (GPT) and triglycerides (TRIG), suggesting normalization of liver function and reduced inflammation. Increases in BUL, CBUN, and the albumin-to-globulin (ALB) ratio reflect recovery in protein metabolism. In females, glucose levels remained elevated, while potassium (K) and chloride (Cl) decreased, indicating mild electrolyte shifts. Overall, these changes appear to be temporary and related to immune response dynamics, with a return to baseline following recovery.

The changes observed in clinical chemistry are not considered toxicologically significant because the values were within the historical control range, and no histopathological correlation was observed in any organ.

#### 3.2.4. Urine Analysis and Gross Pathology

Urine analysis during the treatment period showed decreased specific gravity and increased pH in males and females; however, these changes were significant only in females. During the treatment-free recovery period, both parameters were comparable with those of respective controls. All other urine analysis parameters were comparable, and no histopathological correlation was seen; hence, it was not considered toxicologically adverse (Supplementary Data S3). Gross pathological examination of the treatment groups did not show any lesion of pathological significance compared with the respective control groups (Supplementary Data S4).

#### 3.2.5. Organ Weights

After gross pathology examination, all animals’ organs, viz., liver, kidneys, adrenals, testes/ovaries, thymus, spleen, brain, and heart, were trimmed to remove adhesive tissue and weighed wet. Paired organs were weighed together. Absolute and relative organ weights were calculated for each animal. The organs and tissues were collected at necropsy and fixed in 10% Neutral-Buffered Formalin for subsequent histopathological examination.

Absolute organ weight analysis suggested a non-significant increase in absolute spleen weights in males on day 15 and a non-significant decrease in absolute spleen weight, an increase in absolute kidney weights, and a significant increase in absolute heart weights (G2 and G3) in females (Supplementary Data S5). However, when organ weights relative to their terminal body weights were compared with those of controls, it was observed that in the case of males, there was a non-significant decrease in relative heart weights. In females, relative heart and brain weights only increased significantly in the G2 group (Table 7). The changes observed in both male and female organ weights were recovered at the end of the treatment recovery period, except for a statistically significant increase in the relative thymus weights of G5R females and a significant decrease in the relative liver weights of the G5R male group.

These changes observed in the thymus and spleen might be related to the immunological response to the vaccine and are correlated with follicular hyperplasia on histopathology observation of the spleen (next section). However, No histopathological changes were found in other organs, suggesting that these findings are not considered toxicologically adverse. Additionally, it should also be noted that all changes in organ weights were within the historical range described elsewhere [23].

#### 3.2.6. Histopathology

**Figure 4:**
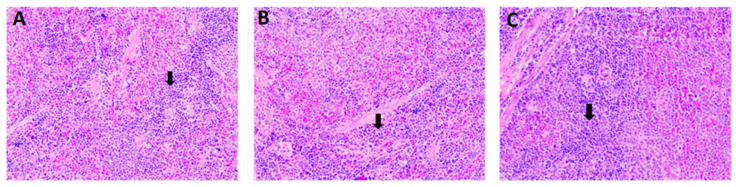
Formation of follicular cell hyperplasia (arrows). (**A**,**B**) in G3 males and (**C**) in G3 female.

To analyze histopathological changes in organs and the injection site, all the representative organs from the control and high-dose groups were processed accordingly and embedded in paraffin. The 3–5 µm thickness sections were cut, stained with hematoxylin-eosin stain, and examined microscopically to analyze the changes (Supplementary Data S6). The analysis revealed no treatment-related histopathological changes in any organs except minimal follicular hyperplasia of the spleen in two males and one female of the G3 group (Figure 4) (Table 8). However, the low and recovery group spleens were comparable to the respective controls.

The spleen is involved in the filtration of pathogens and abnormal cells and facilitates interactions between antigen-presenting cells (APCs) and cognate lymphocytes. APCs in the spleen control how T and B cells react to these antigens in the blood, and follicular hyperplasia is linked to B cells becoming more active [24,25]. Thus, we conclude minimal follicular hyperplasia may have occurred due to the immunization in reflection of a physiological immune reaction that is not toxicologically significant.

### 3.3. Immunogenicity Study in Mice

#### 3.3.1. Mice Safety and Body Weight Transformation

**Figure 5:**
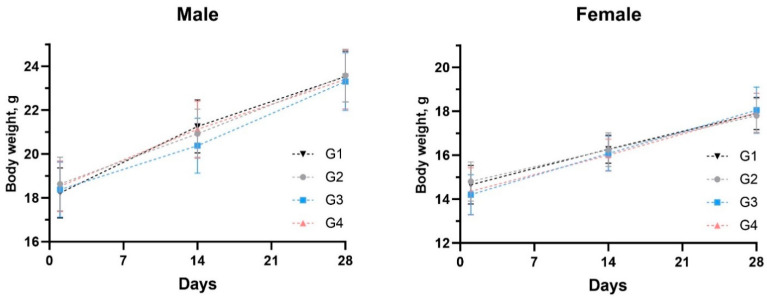
Body weight transformation in mice. The values represent the mean ± SD of all animals per group.

All animals survived throughout the experimental period, and no mortality was observed. No abnormal clinical signs were observed in any animal. After dose administration, local tolerance evaluation revealed no edema or erythema at the injection sites on any day of observation (Days 01, 14, and 28). All the animals gained body weight routinely throughout the experimental period. The body weights of all the treated males and females were comparable to those of control males and females (Figure 5).

#### 3.3.2. Anti-RBD IgG Production

**Figure 6:**
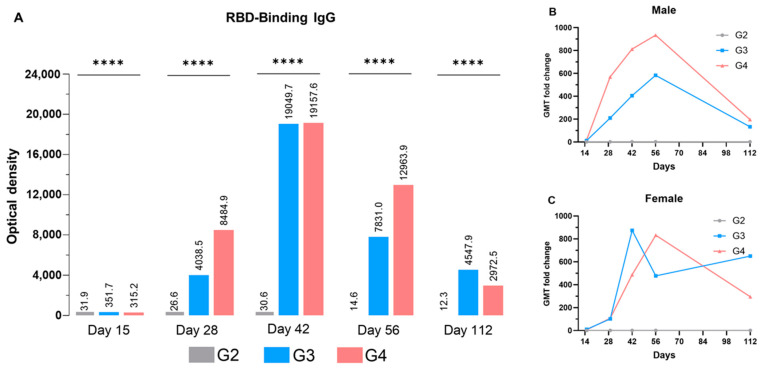
Serological RBD-binding IgG GMT fold change in male and female mice. (**A**) Overall RBD-Binding IgG titer. (**B**) GMT fold change in male mice. (**C**) GMT fold change in female mice. The values represent the mean of all animals per group. *p*-values were analyzed with one-way ANOVA (**** *p* < 0.0001).

The immunogenicity of any vaccine is one of the core qualities that may determine the protective efficacy of that particular candidate. Thus, we studied diverse critical parameters to understand immune response after the RENOVAC vaccine administration. Although protein vaccines, historically, are shown to be less immunogenic than the rest of the platforms, such as viral vectors or attenuated vaccines [26,27], the dimeric RBD vaccine formulated with aluminum hydroxide was shown to induce high rates of specific IgG antibodies on all days, at both low dose (G3) and high dosage (G4) levels (Figure 6).

The animals in the vehicle group exhibited no substantial levels of anti-RBD IgG or neutralizing antibodies. Additionally, no statistically significant differences were observed between the placebo and vehicle control groups for any study experiment at any time. Thus, the PBS group was omitted from the given figures. First, the overall OD values were calculated for each group (Figure 6A), and fold change in geometric mean titers (GMTs) of treatment groups was calculated compared to the G2-placebo group. To do this, the average geometric mean titers were calculated for each animal in the treatment group and then divided by the placebo mean for each corresponding time point and sex. Interestingly, we noticed the male and female mice had varied responses to different vaccine doses (Figure 6B,C). For instance, the rise in GMT was directly proportional to the dose in males on all days except Day 112. In contrast, a 10 µg dose improved anti-IgG production in females at most time points. However, the *t*-test analysis revealed that the differences between G2 and G3 for both sexes were not statistically significant. Yet, the overall rise in GMT level in the G4 group between days 28 and 56 was significantly lower in female mice compared to their male counterparts for the same group. Antibody levels dramatically increased after the first and second booster doses (days 14 and 28) in both males and females. The antibody titer reached its increased peak on day 42 and reduced through day 56 till day 112 at both dose groups. In the case of low-dose females, the peak antibody titer achieved on day 42 was sustained for a prolonged period (up to day 112), suggesting an aggravated antibody response in females compared to males.

The high-level of antibodies observed on day 42 can likely be attributed to the administration route and dose regimen. The intraperitoneal route facilitates more rapid absorption and elicits a stronger immune response, in addition to accommodating higher injection volumes (0.5 mL in our study) compared to the typical 0.05–0.1 mL used in intramuscular injections [16]. Furthermore, the third dose administered in our regimen resulted in a significant increase in antibody titers, in contrast to the more commonly explored two-dose regimens in protein subunit vaccines for COVID-19 [19]. This three-dose approach may have potentiated a more robust and sustained immunological response.

#### 3.3.3. Neutralization Antibody

**Figure 7:**
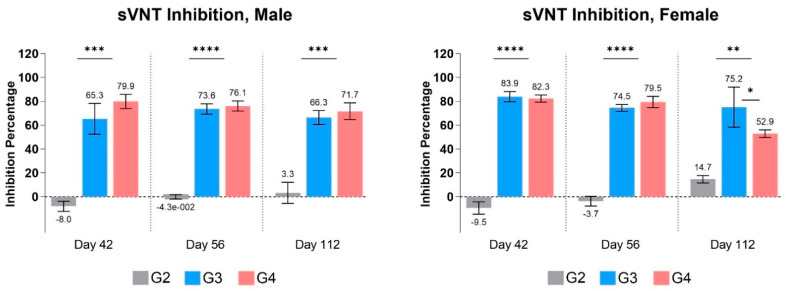
Neutralization efficiency in male and female mice. The values represent the mean ± SEM of all animals per group. *p*-values were analyzed with one-way ANOVA (* *p* < 0.05; ** *p* < 0.01; *** *p* < 0.001; **** *p* < 0.0001).

The neutralization results suggest that the antibodies produced after the vaccination could bind to and neutralize the SARS-CoV-2 antigen conjugate in both males and females at both dose levels (Figure 7). The control groups had no detectable antibodies and showed no significant inhibition, except for minor inhibition in both males and females on day 112. It has also been noticed that neutralizing antibodies are synthesized at slightly higher levels in G4 group male mice compared to G3 group mice. In the case of females, the G3 group had higher levels of NAbs on days 42 and 112, respectively, corresponding to the total anti-RBD IgG produced in female mice (Figure 6C). However, the changes in neutralizing antibody levels were not statistically significant between the G3 and G4 groups, except for female mice on day 112. Several factors might have contributed to the decrease in both specific RBD-binding IgG titers and neutralizing antibodies, high-dose group in comparison to low-dose group in females, including hormonal influences, or possible saturation effects at higher doses, leading to a regulatory feedback mechanism that could suppress antibody production.

Based on the findings from these two studies, we assume a lower dose of the RENOVAC vaccine might be sufficient to induce high levels of target IgGs and provide long-term immunity with a substantial level of neutralizing efficacy. Several other studies show that low amounts of antigen can provide comparable immunogenicity and human protection. For example, a study by Mateus et al. (2021) reported that a low-dose (25 µg) mRNA-1273 (Moderna) vaccine had a comparable effect to a high-dose (100 µg) vaccine in triggering long-lasting T cell immunity in both younger and older patients, even though the concentrations of anti-spike and anti-RBD antibodies were higher at the high-dose regimen [28]. The Phase I and II clinical trials of ZF-2001, a dimeric RBD subunit vaccine, showed that the low-dose vaccine (25 µg/dose) had higher rates of seroconversion and higher levels of SARS-CoV-2-neutralizing antibodies compared to the high-dose vaccine (25 µg/dose). As a result, the lower dose was recommended for subsequent clinical trials [29], and this dosage was only marketed for mass immunization, including in Uzbekistan. However, when administered to mice at 10 µg/dose, the vaccine demonstrated a robust immune response in the development stage [9].

#### 3.3.4. Cellular Immunity

**Figure 8:**
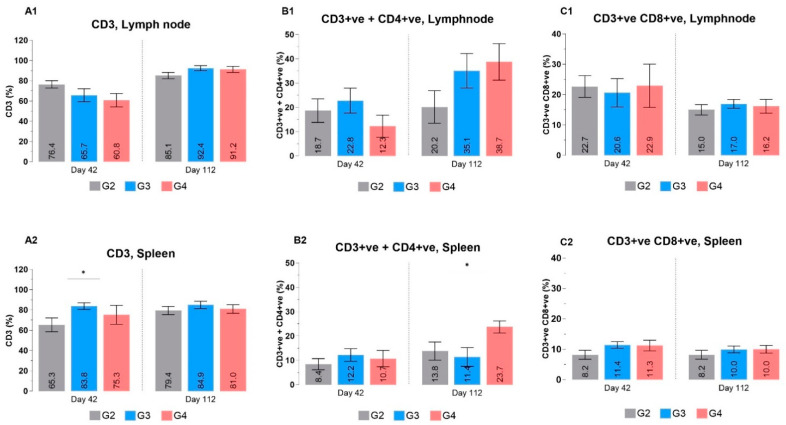
Percentage of CD cells in the lymph node and spleen after immunization. (**A1**,**A2**). The percentage of CD3 cells in lymph node and spleen, respectively. (**B1**,**B2**) The percentage of CD3+ve CD4+ve cells in lymph node and spleen, respectively. (**C1**,**C2**) The percentage of CD3+ve CD8+ve cells in lymph node and spleen, respectively. The values represent the mean ± SEM of all animals per group. *p*-values were analyzed with one-way ANOVA (* *p* < 0.05).

The cellular immune response against the RENOVAC vaccine was evaluated on days 42 and 112 to determine the vaccination’s post-acute and long-term effects on immune cell proliferation. Despite differences in IgG production, males and females exhibited similar trends in the percentage of cells analyzed; however, the response towards the vaccination slightly differed between lymph nodes and spleen. Thus, the values were grouped according to per organ. For example, the CD3+ve cell percentage in the treatment groups was significantly higher in the spleen for day 42, whereas in the lymph node, it showed a decreasing trend (Figure 8).

**Figure 9:**
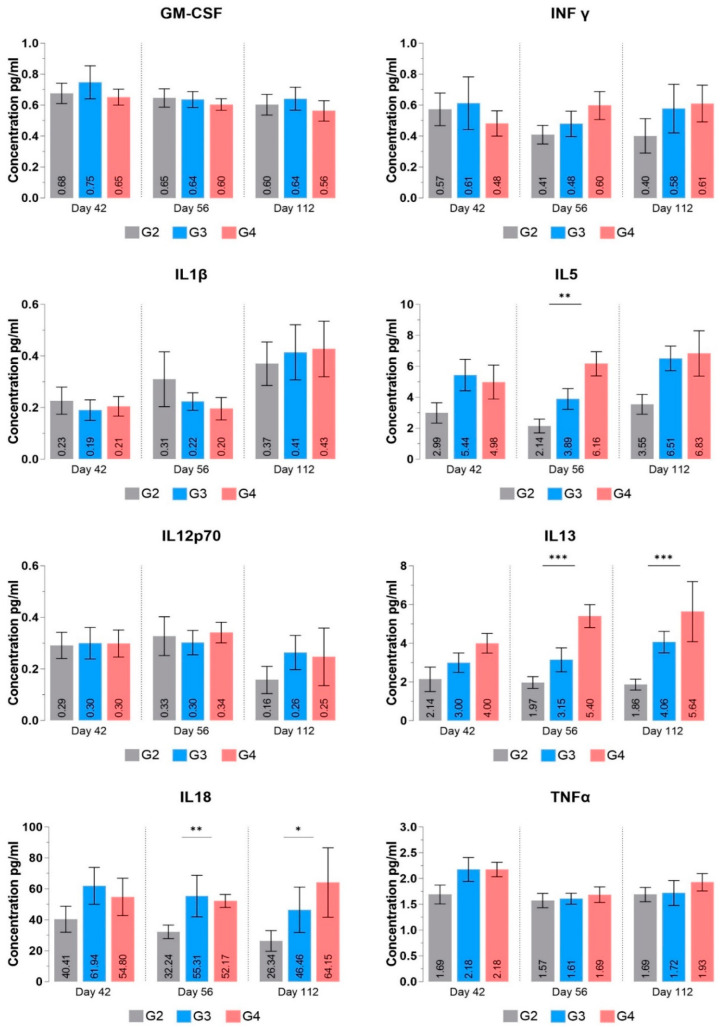
Concentrations of cytokines after the immunization. The values represent the mean ± SEM of all animals per group. *p*-values were analyzed with one-way ANOVA (* *p* < 0.05; ** *p* < 0.01; *** *p* < 0.001).

The percentage CD3+ve + CD4+ve in both spleen and lymph node proportionally reflected cytokines, e.g., IL-5, IFNγ, and GM-CSF, that are produced by Th1 and Th2 cells accordingly upon activation (Figure 9). There is a slight increase in CD3+ve + CD8+ve cells in the spleen for both time points and in lymph nodes on the final day, which might be because of antigen cross-presentation by both major histocompatibility complexes I and II. Antigen cross-presentation is a typical pattern for dendritic cells to internalize exogenous antigens, convert them into antigenic peptides, and deliver these peptides not only with MHC II through the MIIC to CD4+ T cells but also to CD8+ T cells via MHC I through the proteasome and the TAP/ER pathway [30,31].

The reason for no statistically significant differences in CD3+ve + CD4+ve or CD3+ve + CD8+ve cells between the placebo and treatment groups on day 42 might be due to possible stimulation of the immune system earlier, just after the first or second dose of vaccine administration, and finalized proliferation of these cells before. As a result, there might have been no demand for the organisms to keep high loads of effector cells. In general, the initial activation and expansion of CD4+ and CD8+ cells is followed by the death phase, during which around 90% of effector cells are eliminated. Several pathways are involved in the downregulation of cellular response. One mechanism involves TNF and the TNF signaling pathway, which supports induced T cell death to avoid pathogen-associated excessive immune response [32,33]. Higher concentration of TNFα (Figure 9. TNFα) in the vaccination groups on day 42 suggests the possible excessive T cell response could have been eliminated through this path. Finally, upon normalization of TNFα, the effector T cells seem to have been able to expand till day 112. Besides, we tried to retain all data, which prevented changes from being statistically significant because of higher SD values per group. It should also be noted that examining a specific group of T cells formed against RBD protein instead of the general T cell population would allow us to identify changes more clearly, as no considerable amount of specific T cell response would be formed against the antigen in the placebo group.

#### 3.3.5. Cytokine Profile

Vaccination regimes are mostly associated with variations in the profile of cytokines. Changes in cytokine levels govern the immune response against a particular antigen. Further, cytokine levels keep changing as the immune system’s cells continue responding to the antigens.

The purpose of cytokine profiling by multiplex analysis was to determine two pathways: (i) whether the vaccine candidate generates a cytokine storm through the elevation of inflammatory cytokines (e.g., IL1β and IL-6) and (ii) to identify which cytokines are activated in response to vaccination to decipher the pathway of its protective activity.

Our cytokine data depicts that most inflammatory cytokines remain within the historical range; we conclude that the vaccine candidate does not elicit any major inflammatory response that could have resulted in a cytokine storm. Even if there would have been some initial inflammatory response (immediately after injection at day 0, day 14, or day 29, not included in the study), it seems to decline till day 42, and no significant variations have been observed from day 42 till day 112 (Figure 9). Moreover, levels of a few cytokines, including IL-2, IL-4, and IL-6, were undetectable in many samples and did not follow a particular trend. Elevation of IL-6 was previously associated with severe and fatal cases of COVID-19 [34], and another study reported that IL-6 leads to cytokine storm in mice and monkeys after anti-SARS-CoV-2 vaccination [35].

We also observed high levels of IL-18 in samples on days 42, 56, and 112. IL-18 is responsible for initiating the differentiation of Th cells into various subtypes, and its basal levels in serum are already high even under healthy conditions, thus not declining immediately. IL-18 can produce large amounts of IFNγ in the presence of IL-12 [36]. Due to this feature, it has been used as a vaccine adjuvant in many studies and has exhibited potent stimulation effects on Th1 cells [37,38,39,40]. However, the change in IFNγ was statistically insignificant, possibly due to reasons mentioned in the cellular immunity section. Nevertheless, an increasing trend in IFNγ levels reflects changes in IL-18 and CD3+ve + CD4+ve levels over corresponding days, suggesting that RENOVAC vaccination could also trigger Th1 cell responses crucial for the cellular immune system.

The levels of IL-5 and IL-13 in both treatment groups were significantly higher than in the placebo group. These cytokines, secreted by the Th2 subtype of Th cells, activate B-cell-mediated antibody responses and neutrophil activation to facilitate opsonization. IL-4 secretion results in the Th2 subtype, which produces IL-4, IL-5, and IL-13, crucial for activating humoral immune responses mediated via B lymphocytes [41,42,43]. As cytokine profiling data was captured on days 42, 56, and 112, when antibody titers were already very high, it indicated that Th cell differentiation had already occurred. Therefore, maintaining high levels of IL-4, IL-2, or IL-6 was likely unnecessary, leading to their decrease to undetectable levels before day 42. Despite this, the significant increase in other Th2 cell-produced markers suggests IL-4 played a critical role in the early proliferation of naïve CD4+ve cells in T helper cells type 2. The sustained significant levels of IL-13 and IL-5 might have resulted from their essential roles in later B-cell stages, such as self-renewal via a Th2 cell subpopulation [44,45].

Tumor necrosis factor alpha (TNF alpha) is a pleiotropic cytokine produced by macrophages and monocytes responsible for a broad range of signaling events within cells [46]. Initially recognized as a pro-inflammatory molecule, preclinical and clinical studies have since revealed its anti-inflammatory and immunomodulatory effects [47,48,49]. Accordingly, TNF-α suppresses the expansion of T cells, thereby preventing overactive immune responses. It achieves this either by inhibiting the activation of normal T cells or by increasing suppressive cells, such as myeloid-derived suppressor cells or regulatory T cells [32,33,50]. Given these findings, we hypothesize that increased cytokine levels on day 42 or earlier (not checked in the study) may have caused T cell suppression, resulting in minimal changes in T cell numbers.

**Figure 10:**
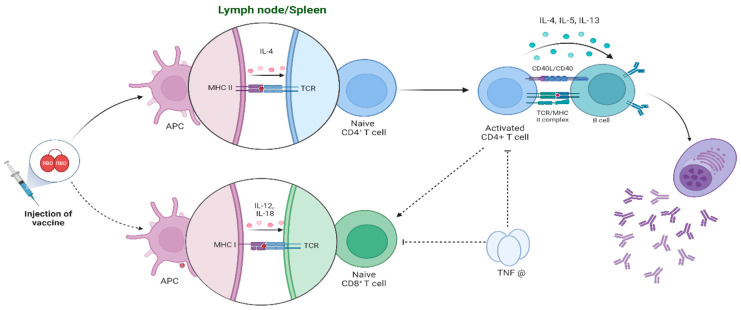
Simplified potential immune system activation mechanism of the RENOVAC vaccine. Dashed arrows represent possible interactions, and undashed arrows represent more likely interactions or paths. Created in BioRender.com.

In summary, the RENOVAC vaccine appears to trigger complex immune responses in mice, leading to high levels of specific inhibitory neutralizing and RBD-specific antibodies. The proposed mechanism involves the activation of T and B cells and corresponding cytokines (Figure 10).

Briefly, the vaccine may act primarily through generating Th2 subpopulations due to CD4+ cell activation. These activated cells stimulate B lymphocytes to become antibody-producing plasma cells. Additionally, CD8+ T cells might also form via activation of Th1 lymphocytes or through cross-presentation of antigens by antigen-presenting cells. Furthermore, TNF-α or other immune-modulatory compounds produced from these stimuli can suppress the activated CD8+ and CD4+ cell populations to restrain excessive immune responses. The dominance of the Th2 response is advantageous for enhancing neutralizing antibody titers, which are vital for immediate protection. Nonetheless, the presence of a Th1 response is essential for viral clearance and sustained immunity [51]. While the strong Th2 response ensures effective antibody-mediated neutralization, the Th1 response, though less dominant, supports long-term immunity and helps clear the virus from the host. This multifaceted approach highlights the importance of a balanced immune response for both immediate and sustained protection.

## 4. Conclusions

We examined the toxicological and immunogenic properties of RENOVAC, a dimer RBD vaccine combined with an alum adjuvant, to assess its preclinical safety and ability to stimulate an immunological response and to understand how the vaccine stimulates the immune system in the body. Based on the results of the present study, it is concluded that the RENOVAC, when administered to Sprague Dawley rats by intramuscular route, was well tolerated up to and including the dose of 25 µg/animal. The changes observed in the spleen and thymus were considered to be related to an immunological response to the vaccine. No toxicologically adverse changes were noted; therefore, the vaccine’s No Observed Adverse Effect Level (NOAEL) is considered to be 25 µg/dose.

Furthermore, when administered to Balb/c mice by intraperitoneal route, the vaccine demonstrated good immunogenicity potential at both low and high doses. However, 10 µg/dose is sufficient to achieve substantial levels of the vaccine-induced anti-RBD and virus-neutralizing antibodies that could be maintained at better rates over a more extended period. The research also revealed that the vaccine primarily triggered an immune response by activating CD4+ helper T cells, leading to the proliferation of Th2 subtype T lymphocytes. This activation of T cells subsequently mediated humoral immunity via the activation of B cells. However, alterations in other indicators like CD8+ cells and IL-18 indicate potential cell-mediated immune activation after the vaccination. This study’s findings support the use of this vaccine in clinical trials.

## Figures and Tables

**Table 1:** Composition of placebo, low-dose, and high-dose vaccine formulations per 0.5 mL.

	RENOVAC: Placebo	RENOVAC: 10 µg	RENOVAC: 25 µg
Active ingredient	0	10 µg	25 µg
20× Alum adjuvant	25 µL	25 µL	25 µL
PBS buffer, pH-7.4	Up to 0.5 mL	Up to 0.5 mL	Up to 0.5 mL

**Table 2:** Experimental design for safety assessment study.

Study Groups	G1	G2	G3	G4 Recovery	G5 Recovery
Treatment	placebo	10 µg	25 µg	placebo	25 µg
Administration	Day 1 and Day 8				
Monitoring Parameters	mortality, clinical signs, local tolerance, detailed clinical examination, body weight, ophthalmoscopic examination, and feed consumption				
Euthanasia	Day 15			Day 43	
Clinical Pathology	hematology, clinical chemistry, urinalysis, gross pathology, organ weights, and histopathological examination				

**Table 3:** Antibodies used for cellular immunity analysis.

Tube No.	Antibody Staining	Ab: Cell Suspension
1	Unstained	-
2	Armenian Hamster IgG isotype control, APC	1:100
3	CD3e monoclonal antibody, APC	1:100
4	Rat IgG2b kappa isotype control, FITC	1:100
5	CD4 monoclonal antibody, FITC	1:100
6	Rat IgG2a kappa isotype control, PE	1:100
7	CD8a monoclonal antibody, PE	1:100
8	Mix of CD3-APC/CD4-FITC/CD8-PE	1:100 each

**Table 4:** Hematology parameters in rats post-RENOVAC vaccine administration.

Parameters		Male					Female				
		G1	G2	G3	G4R	G5R	G1	G2	G3	G4R	G5R
**WBC (10^3^/µL)**	Mean	15.97	16.13	19.02	13.68	10.08	12.63	12.43	12.50	7.07	8.12
	SD	3.72	6.43	5.06	3.80	3.09	4.16	4.16	5.67	1.78	1.06
**RBC (10^6^/µL)**	Mean	7.36	7.30	7.31	8.09	8.54	6.61	6.59	6.98	7.37	7.37
	SD	0.49	0.42	0.39	0.59	0.59	0.43	0.21	0.43	0.49	0.17
**HGB (g/dL)**	Mean	12.65	13.07	12.62	13.38	13.92	12.05	12.50	12.55	12.52	12.92
	SD	0.52	0.45	0.52	0.97	1.08	0.61	0.50	0.82	0.74	0.36
**HCT (%)**	Mean	39.68	40.35	39.27	42.90	44.95	35.55	36.17	37.12	39.13	39.33
	SD	1.61	2.02	1.83	3.11	3.83	1.82	1.32	2.36	2.43	0.96
**MCV (fL)**	Mean	54.02	55.33	53.77	53.05	52.65	53.85	54.87	53.20	53.15	53.37
	SD	1.86	1.81	1.80	1.22	2.08	1.09	1.05	1.18	0.43	0.55
**MCH (pg)**	Mean	17.23	17.95	17.28	16.53	16.28	18.25	18.97 *	18.00	17.03	17.53
	SD	0.78	0.92	0.71	0.60	0.69	0.40	0.39	0.67	0.52	0.50
**MCHC (g/dL)**	Mean	31.90	32.40	32.13	31.20	30.98	33.88	34.55	33.80	32.00	32.87
	SD	0.37	0.77	0.49	0.53	0.43	0.43	0.27	0.73	0.81	0.70
**PLT (10^3^/µL)**	Mean	1067.3	969.3	999.1	801.0	944.3	1023.6	978.0	957.3	870.1	858.5
	SD	156.36	103.71	156.21	160.22	123.38	121.65	116.44	181.60	86.62	120.65
**RETICS (%)**	Mean	0.97	0.80	1.00	1.00	0.93	1.10	1.10	1.07	0.90	0.93
	SD	0.29	0.25	0.36	0.28	0.33	0.37	0.27	0.33	0.37	0.24

Note: WBC—White Blood Cells; RBC—Red Blood Cells; HGB—Hemoglobin; HCT—Hematocrit; MCV—Mean Corpuscular Volume; MCH—Mean Hemoglobin Concentration; MCHC—Mean Corpuscular Hemoglobin Concentration; PLT—Platelet Count; RETICS—Reticulocytes. *N* = 6; SD = standard deviation. G1–G3 day 15, G4R–G5R day 43. Key: * = mean value of group significantly different from placebo control group at *p* < 0.05.

**Table 5:** Differential leukocyte count (%) in rats post-RENOVAC vaccine administration.

Parameters		Male					Female				
		G1	G2	G3	G4R	G5R	G1	G2	G3	G4R	G5R
**Neutrophils**	Mean	22.33	24.00	27.50 *	23.17	24.33	24.67	23.50	25.67	25.50	25.33
	SD	2.25	2.83	3.62	3.37	2.16	3.67	2.81	3.01	2.66	1.63
**Lymphocytes**	Mean	76.17	74.33	71.33 *	75.67	74.17	74.00	75.00	73.00	73.17	73.17
	SD	1.94	2.73	3.27	3.61	2.99	4.20	2.37	3.10	2.79	2.32
**Monocytes**	Mean	0.67	0.83	0.50	0.50	0.67	0.50	0.50	0.50	0.67	0.67
	SD	0.82	0.75	0.84	0.84	0.82	0.84	0.55	0.84	0.82	0.82
**Eosinophils**	Mean	0.83	0.83	0.67	0.67	0.83	0.83	1.00	0.83	0.67	0.83
	SD	0.75	0.75	0.82	0.82	0.75	0.75	0.89	0.75	0.82	0.98
**Basophils**	Mean	0.00	0.00	0.00	0.00	0.00	0.00	0.00	0.00	0.00	0.00
	SD	0.00	0.00	0.00	0.00	0.00	0.00	0.00	0.00	0.00	0.00

Note: *N* = 6; SD = standard deviation. G1–G3 day 15, G4R–G5R day 43. Key: * = mean value of group significantly different from placebo control group at *p* < 0.05.

**Table 6:** Clinical chemistry assessment in rats post-RENOVAC vaccine administration.

Parameters		Male					Female				
		G1	G2	G3	G4R	G5R	G1	G2	G3	G4R	G5R
**GPT (U/L)**	Mean	55.58	58.53	59.88	52.10	36.03 *	53.23	59.12	51.88	49.37	43.72
	SD	13.29	7.35	10.67	10.41	5.10	10.38	9.93	7.52	9.10	8.09
**GOT (U/L)**	Mean	104.73	97.38	111.77	84.57	91.60	83.33	99.13	121.07 *	93.15	108.17
	SD	14.69	26.71	21.05	8.00	12.21	11.98	17.58	39.14	12.69	24.16
**ALP (U/L)**	Mean	341.83	509.83 *	453.67	388.17	313.67	251.83	262.17	328.67	220.50	239.67
	SD	26.83	148.68	100.77	106.15	70.60	65.24	73.20	54.77	93.13	71.01
**BUL (mg/dL)**	Mean	34.47	29.22 *	34.12	27.55	36.05 *	32.23	34.68	36.22	34.52	37.68
	SD	4.81	1.35	3.99	4.63	3.18	6.15	4.28	6.66	2.41	6.08
**CREAT (mg/dL)**	Mean	0.58	0.55	0.60	0.62	0.62	0.57	0.61	0.60	0.62	0.64
	SD	0.04	0.07	0.05	0.05	0.05	0.06	0.04	0.07	0.03	0.03
**GLU (mg/dL)**	Mean	97.10	107.78	115.25	142.62	115.28	101.52	104.13	113.83	91.87	111.92 *
	SD	8.16	22.64	17.58	30.00	13.56	14.11	10.85	17.70	13.04	6.72
**CHOLE (mg/dL)**	Mean	60.50	65.50	64.33	53.83	53.00	71.00	71.67	66.50 *	76.17	71.83
	SD	3.73	5.61	9.93	8.66	5.90	12.49	11.22	6.50	10.21	7.94
**Ca (mg/dL)**	Mean	9.75	9.68	9.98	9.45	9.48	9.68	9.82	9.78	9.60	9.72
	SD	0.41	0.31	0.43	0.52	0.56	0.38	0.45	0.44	0.24	0.33
**ALB (g/dL)**	Mean	2.41	2.33	2.38	2.26	2.42	2.47	2.51	2.56	2.78	2.65
	SD	0.04	0.14	0.10	0.12	0.13	0.14	0.10	0.15	0.14	0.22
**PRO (g/dL)**	Mean	5.98	5.85	6.14	5.90	5.99	6.21	6.05	6.10	6.60	6.64
	SD	0.29	0.46	0.24	0.35	0.44	0.29	0.33	0.37	0.27	0.25
**TRIG (mg/dL)**	Mean	89.52	67.75	71.12	59.17	39.07 *	56.87	50.98	45.95	33.45	29.53
	SD	19.77	12.65	26.33	13.56	15.78	19.36	22.28	15.21	8.32	9.18
**BIT (mg/dL)**	Mean	0.13	0.10	0.11	0.14	0.13	0.11	0.11	0.10	0.10	0.09
	SD	0.05	0.01	0.02	0.04	0.03	0.02	0.02	0.01	0.02	0.02
**ALB/GLO**	Mean	0.68	0.67	0.64	0.62	0.68 *	0.66	0.71	0.73	0.73	0.67
	SD	0.06	0.06	0.06	0.04	0.05	0.06	0.06	0.06	0.03	0.08
**CBUN (mg/dL)**	Mean	16.08	13.63 *	15.92	12.86	16.82 *	15.04	16.19	16.90	16.11	17.59
	SD	2.25	0.63	1.86	2.16	1.48	2.87	2.00	3.11	1.12	2.84
**GLO (g/dL)**	Mean	3.57	3.52	3.76	3.64	3.57	3.75	3.54	3.54	3.82	3.99
	SD	0.31	0.38	0.24	0.28	0.33	0.27	0.28	0.29	0.15	0.21
**Na (mmol/L)**	Mean	138.63	135.38	137.27	138.28	139.35	135.50	139.65	138.98	139.30	138.65
	SD	0.55	7.45	0.56	1.02	1.63	7.19	1.14	1.25	0.72	0.81
**K (mmol/L)**	Mean	4.68	4.65	4.68	4.43	4.66	4.32	4.39	4.55	4.48	3.91 *
	SD	0.25	0.45	0.31	0.32	0.32	0.48	0.13	0.57	0.28	0.12
**Cl (mmol/L)**	Mean	100.88	105.28	100.27	101.77	102.05	105.18	101.55	101.47	103.13	100.77 *
	SD	1.17	9.77	0.98	0.57	1.12	8.57	0.95	1.08	0.74	1.00

Note: GPT—Glutamate Pyruvate Transaminase; GOT—Glutamate Oxaloacetate Transaminase; ALP—Alkaline Phosphatase; BUL—Blood Urea Level; CREAT—Creatinine; GLU—Glucose; CHOLE—Total Cholesterol; Ca—Calcium; ALB—Albumin; PRO—Total Protein; TRIG—Triglycerides; BIT—Total Bilirubin; ALB/GLO—Albumin to Globulin ratio; CBUN—Calculated Blood Urea Nitrogen; GLO—Globulin; Na—Sodium; K—Potassium; Cl—Chloride. *N* = 6; SD = standard deviation. G1–G3 day 15, G4R–G5R day 43. Key: * = mean value of group significantly different from placebo control group at *p* < 0.05.

**Table 7:** Organ weight relative to terminal body weight in rats post-RENOVAC vaccine administration.

Organs/Groups		Male					Female				
		G1	G2	G3	G4R	G5R	G1	G2	G3	G4R	G5R
**Liver**	Mean	4.43	4.27	4.44	4.58	4.01 *	4.03	4.10	4.09	4.21	4.42
	SD	0.50	0.13	0.25	0.46	0.19	0.41	0.45	0.20	0.72	0.54
**Spleen**	Mean	0.33	0.32	0.39	0.29	0.26	0.33	0.33	0.31	0.30	0.32
	SD	0.05	0.09	0.11	0.06	0.07	0.10	0.08	0.11	0.08	0.05
**Heart**	Mean	0.37	0.37	0.36	0.36	0.35	0.35	0.40 *	0.38	0.37	0.38
	SD	0.02	0.03	0.02	0.03	0.02	0.03	0.03	0.02	0.03	0.03
**Thymus**	Mean	0.12	0.10	0.11	0.10	0.11	0.15	0.14	0.16	0.13	0.18 *
	SD	0.03	0.03	0.03	0.03	0.02	0.05	0.02	0.05	0.02	0.03
**Kidneys**	Mean	0.87	0.81	0.84	1.00	0.91	0.78	0.81	0.79	0.94	1.09
	SD	0.13	0.06	0.06	0.11	0.12	0.07	0.06	0.06	0.07	0.19
**Adrenals**	Mean	0.013	0.010	0.014	0.017	0.013	0.024	0.028	0.021	0.032	0.034
	SD	0.004	0.003	0.003	0.004	0.004	0.005	0.006	0.007	0.005	0.011
**Testes/Ovaries**	Mean	1.23	1.15	1.16	1.07	1.02	0.028	0.033	0.030	0.034	0.032
	SD	0.12	0.10	0.06	0.14	0.09	0.002	0.009	0.005	0.009	0.006
**Brain**	Mean	0.79	0.72	0.76	0.72	0.67	0.88	0.95 *	0.91	0.89	0.93
	SD	0.07	0.04	0.07	0.13	0.07	0.06	0.04	0.02	0.09	0.16

Note: *N* = 6; SD = standard deviation. G1–G3 day 15, G4R–G5R day 43. Key: * = mean value of group significantly different from placebo control group at *p* < 0.05.

**Table 8:** Occurrence of follicular hyperplasia in spleen.

Sex	Males					Females				
Group	G1	G2	G3	G4 R	G5 R	G1	G2	G3	G4 R	G5 R
Spleen	0/6	0/6	2/6	0/6	0/6	0/6	0/6	1/6	0/6	0/6

## Data Availability

Data is presented within the article, and Supplementary Data is attached to the article.

## Supplementary Materials

The following supporting information can be downloaded at: https://www.mdpi.com/article/10.3390/vaccines12121420/s1, Supplementary Data S1: Body Weight Gain (gm). Supplementary Data S2: Feed Consumption (gm). Supplementary Data S3: Qualitative Urinalysis. Supplementary Data S4: Gross Pathology Observations. Supplementary Data S5: Absolute Organ Weight (gm). Supplementary Data S6: Histopathology Images Female & Male.
