# Supplementary material for: Development, Pre-Clinical Safety, and Immune Profile of RENOVAC—A Dimer RBD-Based Anti-Coronavirus Subunit Vaccine
Source: Vaccines (Basel). 2024 Dec 17;12(12):1420. doi: 10.3390/vaccines12121420 (PMC11680381; doi:10.3390/vaccines12121420)
Supplement: Supplementary file 1 [file vaccines-12-01420-s001.zip › Ethical Approval-IAEC-23-002.pdf]

Form B (per rule 8 (a)* for Submission of Research Protocol (s)

Application for Permission for Animal Experiments

Application to be submitted to the CPCSEA, New Delhi after approval of Institutional Animal Ethics Committee (IAEC)

Section -I

1.	Name and address of the establishment	PRADO Private Limited, Pune
2.	Registration number and date of registration	1723/PO/ReBiBt/S/13/CPCSEA dated May 06, 2017
3.	Name, address and Registration number of breeder from which animals acquired (or to be acquired) for experiments mentioned in parts B and C.	CPCSEA approved organizations
4.	Place where the animals are presently kept (or proposed to be kept)	Animal Research Facility, PRADO Pvt. Ltd., Pune.
5.	Place where the experiment is to be performed (please provide CPCSEA reg. Number)	Animal Research Facility, PRADO Pvt. Ltd., Pune. (1723/PO/ReBiBt/S/13/CPCSEA)
6.	Date and Duration of experiment	Within 1 year after ethics committee's approval. Duration of experiment- 3-5 months.
7.	Type of research involved (Basic Research /Educational /Regulatory/Contract Research)	Contract Research and Regulatory

Date: Feb 04, 2023

Sandhya MVS Feb 04, 2023

Signature

Place: Pune

Name and Designation of Investigator

(Dr. Sandhya MVS, Study Director)

Section –II
Protocol form for research proposals to be submitted to the Institutional Animal Ethics Committee/ CPCSEA, for new experiments or extensions of ongoing experiments using animals.

1. Project / Dissertation / Thesis Title : **Evaluation of Immunogenicity of d-RBD protein vaccine in Balb/c Mice**
2. Principal Investigator
   a. Name : *Dr. Sandhya MVS*
   b. Designation : *Study Director*
   c. Dept/Div/Lab : *In vitro Genotoxicity*
   d. Telephone number : *91-9989838744*
   e. E-mail Id : *ivg@pradopreclinical.com*
   f. Experience in Lab animal experimentation : *6 years*
3. List of all individuals authorized to conduct procedures under this proposal.

Sr. No.	Name	Designation	Department	Telephone No.	E-mail Id	Experience in Lab animal experimentation
1.	*Ankush Dewle*	*Deputy Study Director*	*IVG*		*ankush.dewle@pradopreclinical.onmicrosoft.com*	*4 years*
2.	*Dr. Noopur Halmare*	*ARF in-charge*	*ARF*	*9989838744*	*arf@pradopreclinical.com*	*3 years*
3.	*Dr. Pradhnya Choudhari*	*Veterinary Pathologist*	*PAT*		*pathology@pradopreclinical.com*	*2 years 1 month*
4	*Pooja Khavare*	*Study Personnel*	*IVG*		-	*8 months*

4. Funding Source / Proposed Funding Source with complete address (Please attach the proof)
   Respective Sponsors
5. Duration of the project
   a. Date of initiation (Proposed) *- Feb, 2023*
   b. Date of completion (Proposed) *- Feb, 2024*

6. Describe details of study plan to justify the use of animals (Enclose Annexure)

Mice (Balb/c) (6-8 weeks)	Male	Female
G1- Vehicle Control	25	25
G2- Adjuvant Control	25	25
G3- Low Dose	25	25
G4- Mid Dose	25	25

The study will be conducted following New Drugs and Clinical Trials Rules, 2019 and World Health Organization Technical Report Series, No. 927, 2005 and European Medicines Agency, Guideline EMA/CHMP/VWP/141697/2009, June 2010. Animals will be acclimatized at least

for 5 days and will be randomized on the basis of body weight into different treatment groups and control groups (Vehicle Control, Adjuvant Control, Low and Mid dose groups). Experimental groups will contain 25 animals/sex/group. During acclimatization and experimental period standard mice feed and water will be provided ad-libitum, except during fasting. The test item or vehicle will be administered on Day 0 followed by 1st booster dose on Day 14 and 2nd booster dose on Day 28 to animals via route as per the study plan requirements. The study will be divided into following 4 experimental phases and all the experiments will be performed utilizing the same animals;

- *S-RBD IgG detection by ELISA*
- *Cytokine profiling by multiplexing*
- *Cellular Immunity (CD3, CD4, CD8) in spleen and lymph node*
- *Neutralizing Antibody Titer by ELISA*

All animals will be observed for clinical signs, body weight change and feed consumption during experimental period. Blood samples (without any anticoagulant) and organs from the respective animals from each group will be collected. The serum samples will be separated on Day 15, 29, 42, 56 and 112 (5/sex/group) and organs will be collected on Day 42, 56 and 112 and will be processed as per the study plan requirements. The carcasses will be disposed of by incineration (Life secure Environmental Solutions Pvt. Ltd. Pune).

7. Animals required

a. Species and Strain - *Mice (Balbc)*

b Age and Weight - *6 - 8 weeks / 20 to 40 gm*

c. Gender - *Male and Female*

d. Number to be used (Year-wise breakups and total figures needed to be given in tabular form)

No of animals / Study (As per OECD and New Drugs and Clinical Trials Rules, 2019)	No. of Toxicity Studies per year	Total number of animals
200 (25 Animals/sex/group 4 groups)	*01*	*200*

e. Number of days each animal will be housed – *112 days.*

8. Rationale for animal usage

a. Why is animal usage necessary for these studies?

Multiple assays are required to measure immune responses to demonstrate the immunogenicity, efficacy, exposure, safety and provide confidence that the candidate vaccine is at least capable of eliciting a robust immune response.

b. Whether similar study has been conducted on *in vitro* models? If yes, describe the leading points to justify the requirement of animal experiment. *No*

c. Why are the particular species selected?

Rat is one of the acceptable rodent species to regulatory bodies and widely used model for evaluating toxic effects of d-RBD vaccine.

d. Why is the estimated numbers of animals essential?

This is minimum number of animals required as per regulatory guidelines.

e. Are similar experiments conducted in the past in your establishment? *No*

f. If yes, justify why new experiment is required? *NA*

g. Have similar experiments been conducted by any other organization in same or other *in vivo* models? If yes, enclose the reference:

As per Sponsor's communication, no previous experiments have been conducted in past.

9. Describe the procedures in detail:

a. Describe all invasive and potentially stressful non-invasive procedures that animals will be subjected to in the course of the experiments).

Animals will be dosed via route as per the study plan requirement. Animals will be dosed via intramuscular route and dosing will be performed by trained persons following aseptic conditions. The in-house SOP (PRADO/ARF/SOP/007) of PRADO Pvt. Ltd will be used for the same. (Refer Annexure II)

b. Furnish details of injections schedule:

Substances	:	*d-RBD vaccine products*
Doses	:	*Will be decided based on the regulatory guidelines, literature review or as per study plan's requirement*

c. Sites : : *Intramuscular*

Volumes : : *Will be decided based on the sponsor requirement*

Blood withdrawal Details: : *Yes (under the dosage of ketamine at dose rate of 120 mg/kg and Xylazine 16 mg/kg at dose rate of 0.1 ml per 10g of body weight by intraperitoneal injection. Anesthesia will be performed by trained persons following aseptic conditions.*

d. Volumes : : *0.5 ml*

e. Sites : : *Retro-orbital Sinus*

Radiations (Dosage and schedules) : *NA*

Nature of compound/Broad Classification of drug/NCE: *Pharmaceutical*

10 Does the protocol prohibit use of anesthetic or analgesic for the conduct of painful procedures? If yes, justify. *No*

11 Will survival surgery be done? *No*

If yes, the following to be described

a. List and describe all surgical procedures (including methods of asepsis): *NA*

b. Names, qualifications and experience levels of personnel involved: *NA*

c. Describe post-operative care: *NA*

d. Justify if major survival surgery is to be performed more than once on a single animal: *NA*

12 Describe post-experimentation procedures

a. Scope for Reuse: *No*

b. Rehabilitation (Name and Address, where the animals are proposed to be rehabilitated): *NA*

Animals will not be re-used or rehabilitated as organs will be collected at different time points

c. Describe method of euthanasia (If required in the protocol):

Animals will be euthanised by Thiopental Sodium at 150 mg/kg body weight through intraperitoneal route as per the in-house SOP (PRADO/PAT/SOP/007).

d. Method of carcass disposal after euthanasia:

Animal carcass will be send for Incineration to MPCB approved and registered vendor (Life Secure Enterprises, Pune).

13 Describe animal transportation methods if extra-institutional transport is envisaged.

Animal will be transported as per CPCSEA guidelines using air-conditioned vehicles. In house SOP will be followed (PRADO/ARF/SOP/008).

14 Use of hazardous agents (use of recombinant DNA-based agents or potential human pathogens

requires documented approval of the Institutional Biosafety Committee (IBSC). For each category, the agents and the biosafety level required, appropriate therapeutic measures and the mode of disposal of contaminated food, animal wastes and carcasses must be identified). If, your project involved use of any of the below mentioned agent, attach copy of the approval certificates of the respective agencies:

a. Radionucleotides (AERB): *NA*
b. Microorganisms / Biological infectious Agents (IBSC): *NA*
c. Recombinant DNA (RCGM): *NA*
d. Any other Hazardous Chemical / Drugs: *NA*

Investigator's declaration

1. I certify that the research proposal submitted is not unnecessarily duplicative of previously reported research.

2. I certify that I am qualified and have experience in the experimentation in the animals.

3. For procedures listed under item 10, I certify that I have reviewed the pertinent scientific literature and have found no valid alternative to any procedure described herein which may cause less pain or distress.

4. I will obtain approval from the IAEC/CPCSEA before initiating any significant changes in this study.

5. I certify that performance of experiment will be initiated only upon review and approval of scientific intent by appropriate expert body (Institutional Scientific Advisory Committee / funding agency /other body).

6. I certify that I will submit appropriate certification of review and concurrence for studies mentioned in point 14.

7. I shall maintain all the records as per format (Form D) and submit to Institutional Animal Ethics Committee (IAEC).

8. I certify that, I will not initiate the study before approval from IAEC/ CPCSEA received in writing. Further, I certify that I will follow the recommendations of IAEC/ CPCSEA.

9. I certify that I will ensure the rehabilitation policies are adopted (wherever required).

Date: Feb 04, 2023.

Signature

Name of Investigator

(Dr. Sandhya MVS)

Certificate

This is to certify that the project proposal no. **IAEC-23-002** entitled **Evaluation of Immunogenicity of d-RBD protein vaccine in Balb/c Mice** submitted by **Dr. Sandhya MVS** has been approved/recommended by the IAEC of **PRADO Pvt. Ltd., Pune** in its meeting dated **Feb 04, 2023** and has been sanctioned **200 mice** under this proposal for a duration of next **twelve** months.

Authorized by	Name	Signature	Date
Chairman	Ila Wangikar	[signature]	Feb 04, 2023
Member Secretary	Dr. Noopur Halmare	[signature]	Feb 04, 2023
Link Nominee of CPCSEA (As per the written consent from Main Nominee)	Dr. Shivaji Gade	[signature]	04/02/23

(Kindly make sure that minutes of the meeting duly signed by all the participants are maintained by Office).

Annexure I: Blood and Organ collection details

Group No.	Collection	Day of Collection 15	29	42	56	112
G1	Blood	X	X	X	X	X
		X	X	X	X	X
	Spleen and Lymph			X	X	X
				X	X	X
G2	Blood	X	X	X	X	X
		X	X	X	X	X
	Spleen and Lymph			X	X	X
				X	X	X
G3	Blood	X	X	X	X	X
		X	X	X	X	X
	Spleen and Lymph			X	X	X
				X	X	X
G4	Blood	X	X	X	X	X
		X	X	X	X	X
	Spleen and Lymph			X	X	X
				X	X	X
