# Supplementary material for: Development, Pre-Clinical Safety, and Immune Profile of RENOVAC—A Dimer RBD-Based Anti-Coronavirus Subunit Vaccine
Source: Vaccines (Basel). 2024 Dec 17;12(12):1420. doi: 10.3390/vaccines12121420 (PMC11680381; doi:10.3390/vaccines12121420)
Supplement: Supplementary file 1 [file vaccines-12-01420-s001.zip › Ethical Approval-IAEC-23-003.pdf]

Form B (per rule 8(a)* for Submission of Research Protocol (s)

Application for Permission for Animal Experiments

Application to be submitted to the CPCSEA, New Delhi after approval of Institutional Animal Ethics Committee (IAEC)

Section -I

1.	Name and address of the establishment	PRADO Private Limited, Pune
2.	Registration number and date of registration	1723/PO/ReBiBt/S/13/CPCSEA dated May 06, 2017
3.	Name, address and Registration number of breeder from which animals acquired (or to be acquired) for experiments mentioned in parts B and C.	CPCSEA approved organizations
4.	Place where the animals are presently kept (or proposed to be kept)	Animal Research Facility, PRADO Pvt. Ltd., Pune.
5.	Place where the experiment is to be performed (please provide CPCSEA reg. Number)	Animal Research Facility, PRADO Pvt. Ltd., Pune. (1723/PO/ReBiBt/S/13/CPCSEA)
6.	Date and Duration of experiment	Within 1 year after ethics committee's approval. Duration of experiment- 2-3 months.
7.	Type of research involved (Basic Research /Educational /Regulatory/Contract Research)	Contract Research and Regulatory

Date: Feb 04, 2023

Place: Pune

cghogale

Signature

Name and Designation of Investigator

(Mr. Chetan Ghogale, Study Director)

Section –II
Protocol form for research proposals to be submitted to the Institutional Animal Ethics Committee/ CPCSEA, for new experiments or extensions of ongoing experiments using animals.

1. Project / Dissertation / Thesis Title : **Repeated Dose Toxicity Along With the Recovery Group in RBD Protein Vaccine in Rats**

2. Principal Investigator
   a. Name : *Mr.Chetan Ghogale*
   b. Designation : *Study Director*
   c. Dept/Div/Lab : *Toxicology*
   d. Telephone number : *7030525535*
   e. E-mail Id : *Chetan.ghogale@pradopreclinical.com*
   f. Experience in Lab animal experimentation : *15 Years*

3. List of all individuals authorized to conduct procedures under this proposal.

Sr. No.	Name	Designation	Department	Telephone No.	E-mail Id	Experience in Lab animal experimentation
1.	*Ms. Sarika Suryatale*	*Study Personnel*	*Toxicology*	*7030525535*	*sarika.suryatale@pradopreclinical.com*	*6 Years*
2.	*Mr. Amol Kamble*	*Study Personnel*	*Toxicology*		*amol.kamble@pradopreclinical.com*	*7 Years*
3.	*Ms. Sarika Shelke*	*Study Personnel*	*Toxicology*		*sarika.shelke@pradopreclinical.com*	*2 Years*
4.	*Dr. Pradhnya Choudhari*	*Study Pathologist*	*Pathology*		*pathology@pradopreclinical.com*	*2 years 1 months*
5.	*Dr.Noopur Halmare*	*Veterinarian*	*Animal Research Facility*		*arf@pradopreclinical.com*	*3 years*

4. Funding Source / Proposed Funding Source with complete address (Please attach the proof)
   Respective Sponsors

5. Duration of the project
   a. Date of initiation (Proposed) - *Feb, 2023*
   b. Date of completion (Proposed) - *Feb, 2024*

6. Describe details of study plan to justify the use of animals (Enclose Annexure)
   The study will be conducted following OECD guideline (TG 407) and Drugs and Clinical Trials Rules, 2019. Animals will be acclimatized at least for 5 days and will be randomized on the basis of body weight into control group and control recovery group and treatment groups and treatment recovery groups based on study plan's requirements. Experimental groups will contain 6 animals/sex/group. During acclimatization and experimental period standard rat feed and water will be provided ad-lib, except during fasting. The test item or vehicle will be

administered daily to animals via intramuscular route. All animals will be observed for clinical signs, body weight change and feed consumption during experimental period. After completion of treatment and/or recovery periods, animals will be fasted overnight and blood samples (2 ml) will be collected for haematology and clinical chemistry analysis. Urine will be collected for urine analysis. Animals will be euthanized and observations such as gross pathology, organ weights and histopathological evaluation will be performed as per the study plan requirements. The carcasses will be disposed of by incineration (Life Secure Enterprises, Pune). Humane end point: During conduct of the study if the animal(s) are found in moribund state or in stress or distress, unable to access to feed/water animals will be humanely euthanized by CO_2 asphyxiation (PRADO/PAT/SOP/001 and PRADO/PAT/SOP/007).

Immunogenicity experiments Details of Requirements:

Species	Rats
**Number of animals**	72 (6/sex/group) 6 groups
**Adjuvant Control**	12 (6 males + 6 females) control and 12 (6 males + 6 females) recovery
**Test Vaccine RBD (Dose 1)**	12 (6 males + 6 females) – test vaccine Low dose and 12 (6 males + 6 females) - recovery
**Test Vaccine RBD (Dose 2)**	12 (6 males + 6 females)- test vaccine Mid Dose test and 12 (6 males + 6 females) - recovery

7. Animals required

a. Species and Strain - *Rat, Sprague Dawley*

b Age and Weight - *5 - 7 weeks / 130-220 gm*

c. Gender - *Male and Female*

d. Number to be used (Year-wise breakups and total figures needed to be given in tabular form)

No of animals / Study (As per OECD and New Drugs and Clinical Trials Rules, 2019)	No. of Toxicity Studies per year	Total number of animals
72 (6 Animals/sex/group – 6 groups)	*2*	*144*

e. Number of days each animal will be housed – 70 days

8. Rationale for animal usage

a. Why is animal usage necessary for these studies?
Repeated dose toxicity study is more accurate way of estimating No Observed Adverse Effect level (NOAEL) of chemicals / healthcare products which help in selection of dosage for long term toxicity studies. The data also support in selection of safe dose for human exposure / trials. No alternate in vitro models available for this purpose.

b. Whether similar study has been conducted on in vitro models? If yes, describe the leading points to justify the requirement of animal experiment. *No*

c. Why are the particular species selected?
Rat is one of the acceptable rodent species to regulatory bodies and widely used model for evaluating toxic effects of chemicals and health care products.

d. Why is the estimated numbers of animals essential?
This is minimum number of animals required as per regulatory guidelines.

e. Are similar experiments conducted in the past in your establishment? *No*

f. If yes, justify why new experiment is required? *NA*

g. Have similar experiments been conducted by any other organization in same or other *in vivo* models? If yes, enclose the reference:
As per Sponsor's communication, no previous experiments have been conducted in past.

9. Describe the procedures in detail:

a. Describe all invasive and potentially stressful non-invasive procedures that animals will be subjected to in the course of the experiments).
Animals will be dosed via intramuscular route. Dose administration by IM will be performed by trained persons following aseptic conditions and as per in-house SOP (PRADO/ARF/SOP/007) of PRADO Pvt. Ltd will be used for the same.

b. Furnish details of injections schedule:

Substances	:	*RBD Protein Vaccine*
Doses	:	*Based on the regulatory guidelines, literature review or as per study plan's requirement*
Sites :	:	*Intramuscular*
Volumes :	:	*Will be decided based on the sponsor requirement*
c. Blood withdrawal Details:	:	*Yes (under 30% v/v mild isoflurane anaesthesia in propylene glycol.)*
Volumes :	:	*2 ml*
Sites :	:	*Retro-orbital Sinus*
d. Radiations (Dosage and schedules)	:	*NA*

e. Nature of compound/Broad Classification of drug/NCE: *Pharmaceutical.*

10 Does the protocol prohibit use of anaesthetic or analgesic for the conduct of painful procedures? If yes, justify.
No, the protocol does not prohibit use of anaesthetic or analgesic for the conduct of painful procedures.

11 Will survival surgery be done? *No*
If yes, the following to be described

a. List and describe all surgical procedures (including methods of asepsis): *NA*

b. Names, qualifications and experience levels of personnel involved: *NA*

c. Describe post-operative care: *NA*

d. Justify if major survival surgery is to be performed more than once on a single animal: *NA*

12 Describe post-experimentation procedures

a. Scope for Reuse: *No*

b. Rehabilitation (Name and Address, where the animals are proposed to be rehabilitated):
NA. Animals will not be re-used or rehabilitated as organs will be collected for histopathology.

c. Describe method of euthanasia (If required in the protocol):
At the end of Experiment animals will be sacrificed by CO_2 *asphyxiation as per the in-house SOP (PRADO/PAT/SOP/007).*

d. Method of carcass disposal after euthanasia:
Animal carcass will be send for Incineration to MPCB approved and registered vendor (Life

Secure Enterprises, Pune).

13 Describe animal transportation methods if extra-institutional transport is envisaged.
Animals will be transported as per CPCSEA guidelines using air-conditioned vehicles. In-house SOP will be followed. (PRADO/ARF/SOP/008).

14 Use of hazardous agents (use of recombinant DNA-based agents or potential human pathogens requires documented approval of the Institutional Biosafety Committee (IBC). For each category, the agents and the biosafety level required, appropriate therapeutic measures and the mode of disposal of contaminated food, animal wastes and carcasses must be identified). If, your project involved use of any of the below mentioned agent, attach copy of the approval certificates of the respective agencies:

a. Radionucleotides (AERB): *NA*
b. Microorganisms / Biological infectious Agents (IBSC): *NA*
c. Recombinant DNA (RCGM): *NA*
d. Any other Hazardous Chemical / Drugs: *NA*

Investigator's declaration

1. I certify that the research proposal submitted is not unnecessarily duplicative of previously reported research.

2. I certify that I am qualified and have experience in the experimentation in the animals.

3. For procedures listed under item 10, I certify that I have reviewed the pertinent scientific literature and have found no valid alternative to any procedure described herein which may cause less pain or distress.

4. I will obtain approval from the IAEC/CPCSEA before initiating any significant changes in this study.

5. I certify that performance of experiment will be initiated only upon review and approval of scientific intent by appropriate expert body (Institutional Scientific Advisory Committee / funding agency /other body).

6. I certify that I will submit appropriate certification of review and concurrence for studies mentioned in point 14.

7. I shall maintain all the records as per format (Form D) and submit to Institutional Animal Ethics Committee (IAEC).

8. I certify that, I will not initiate the study before approval from IAEC/ CPCSEA received in writing. Further, I certify that I will follow the recommendations of IAEC/ CPCSEA.

9. I certify that I will ensure the rehabilitation policies are adopted (wherever required).

Date: Feb 04, 2023

cgghogale
Signature

Name of Investigator
(*Mr. Chetan Ghogale*)

Certificate

This is to certify that the project proposal no. **IAEC-23-003** entitled **Repeated Dose Toxicity Along With the Recovery Group in RBD Protein Vaccine in Rats** submitted by Dr. / **Mr.** / Ms. **Chetan Ghogale** has been approved/recommended by the IAEC of **PRADO Pvt. Ltd., Pune** in its meeting dated **Feb 04, 2023** and has been sanctioned **144 rats** under this proposal for a duration of next **twelve** months.

Authorized by	Name	Signature	Date
Chairman	Ila Wangikar	[signature]	Feb 04, 2023
Member Secretary	Dr. Noopur Halmare	[signature]	Feb 04, 2023
Link Nominee of CPCSEA (As per the written consent from Main Nominee)	Dr. Shivaji Gade	[signature]	04/02/23

(Kindly make sure that minutes of the meeting duly signed by all the participants are maintained by Office)
