# Supplementary material for: Development, Pre-Clinical Safety, and Immune Profile of RENOVAC—A Dimer RBD-Based Anti-Coronavirus Subunit Vaccine
Source: Vaccines (Basel). 2024 Dec 17;12(12):1420. doi: 10.3390/vaccines12121420 (PMC11680381; doi:10.3390/vaccines12121420)
Supplement: Supplementary file 1 [file vaccines-12-01420-s001.zip › Supplementary Data S1.pdf]

Supplementary Data S1: Body Weight Gain (gm)

Sex: Male

Mean/ SD/N	Body Weight Gain	
	7	14
**G1 Placebo Control µg/animal**		**Dose: 0**
**Mean**	18.42	43.50
**SD**	8.67	18.56
**N**	6	6
**G2 Low Dose 10µg/animal**		**Dose:**
**Mean**	24.92	45.25
**SD**	8.88	13.34
**N**	6	6
**G3 High Dose 25µg/animal**		**Dose:**
**Mean**	21.83	40.25
**SD**	3.39	11.96
**N**	6	6

Mean/ SD/N	Body Weight Gain					
	7	14	21	28	35	42
**G4 Placebo Control-R**						**Dose: 0 [illegible]**
**Mean**	25.92	44.25	74.58	93.33	108.50	127.92
**SD**	11.41	16.04	24.63	26.65	31.80	41.79
**N**	6	6	6	6	6	6
**G5 High Dose –R**						**Dose : 25 µg/animal**
**Mean**	21.00	41.92	68.50	90.83	106.00	122.17
**SD**	9.93	12.76	19.20	19.72	23.54	19.72
**N**	6	6	6	6	6	6

Note: N = number of animals; SD = Standard Deviation

Supplementary Data S1 (cont.): **Body Weight Gain (gm)**

Sex: Female

Mean/ SD/N	Body Weight Gain	
	7	14
**G1 Placebo Control μg/animal**		**Dose: 0**
**Mean**	7.75	19.00
**SD**	3.31	3.24
**N**	6	6
**G2 Low Dose 10μg/animal**		**Dose:**
**Mean**	8.50	15.67
**SD**	3.62	5.52
**N**	6	6
**G3 High Dose 25μg/animal**		**Dose:**
**Mean**	9.17	19.58
**SD**	2.68	3.60
**N**	6	6

Mean/ SD/N	Body Weight Gain					
	7	14	21	28	35	42
**G4 Placebo Control-R**						**Dose: 0** [illegible]
**Mean**	7.33	16.33	27.17	39.50	49.50	65.25
**SD**	3.49	5.40	5.85	6.40	7.04	5.35
**N**	6	6	6	6	6	6
**G5 High Dose –R**						**Dose : 25** [illegible]
**Mean**	10.08	18.08	28.17	39.50	48.58	58.92
**SD**	2.11	5.48	6.37	9.95	10.64	9.85
**N**	6	6	6	6	6	6

Note: N = number of animals; SD = Standard Deviation.
