# Supplementary material for: Development, Pre-Clinical Safety, and Immune Profile of RENOVAC—A Dimer RBD-Based Anti-Coronavirus Subunit Vaccine
Source: Vaccines (Basel). 2024 Dec 17;12(12):1420. doi: 10.3390/vaccines12121420 (PMC11680381; doi:10.3390/vaccines12121420)
Supplement: Supplementary file 1 [file vaccines-12-01420-s001.zip › Supplementary Data S2.pdf]

Supplementary Data S2: Feed Consumption (gm)

Sex: Male

**Mean/ SD/N**	**Feed Consumption**	
	**7**	**14**
**G1 Placebo Control Dose: 0 μg/animal**		
**Mean**	292.00	375.00
**SD**	2.12	6.36
**N**	6	6
**G2 Low Dose Dose: 10μg/animal**		
**Mean**	297.75	374.00
**SD**	5.30	7.07
**N**	6	6
**G3 High Dose Dose: 25μg/animal**		
**Mean**	292.25	368.25
**SD**	25.10	25.81
**N**	6	6

**Mean/ SD/N**	**Feed Consumption**					
	**7**	**14**	**21**	**28**	**35**	**42**
**G4 Placebo Control-R Dose: 0 [illegible]**						
**Mean**	292.00	375.00	383.50	381.00	381.25	382.50
**SD**	14.14	12.73	8.49	8.49	1.77	2.12
**N**	6	6	6	6	6	6
**G5 High Dose –R Dose : 25 [illegible]**						
**Mean**	287.50	386.00	388.00	382.25	383.75	384.50
**SD**	9.19	2.83	2.12	3.18	8.13	7.07
**N**	6	6	6	6	6	6

Note: No. = number of animals

Supplementary Data S2 (Contd.): Feed Consumption (gm)

Sex: Female

**Mean/ SD/N**	**Feed Consumption**	
	**7**	**14**
**G1 Placebo Control Dose: 0 µg/animal**		
**Mean**	296.75	323.50
**SD**	3.18	26.87
**N**	6	6
**G2 Low Dose Dose: 10µg/animal**		
**Mean**	294.00	320.00
**SD**	1.41	3.54
**N**	6	6
**G3 High Dose Dose: 25µg/animal**		
**Mean**	295.25	330.50
**SD**	6.72	0.71
**N**	6	6

**Mean/ SD/N**	**Feed Consumption**					
	**7**	**14**	**21**	**28**	**35**	**42**
**G4 Placebo Control-R Dose: 0 [illegible]**						
**Mean**	275.00	331.75	370.50	362.50	363.75	365.75
**SD**	6.36	38.54	6.36	4.95	6.01	6.01
**N**	6	6	6	6	6	6
**G5 High Dose –R Dose : 25 [illegible]**						
**Mean**	276.25	340.00	370.00	364.00	368.00	371.50
**SD**	2.47	5.66	9.90	2.83	2.12	2.12
**N**	6	6	6	6	6	6

Note: No.= number of animals
