# Supplementary material for: Development, Pre-Clinical Safety, and Immune Profile of RENOVAC—A Dimer RBD-Based Anti-Coronavirus Subunit Vaccine
Source: Vaccines (Basel). 2024 Dec 17;12(12):1420. doi: 10.3390/vaccines12121420 (PMC11680381; doi:10.3390/vaccines12121420)
Supplement: Supplementary file 1 [file vaccines-12-01420-s001.zip › Supplementary Data S3.pdf]

Supplementary Data S3 Qualitative Urinalysis

Sex: Male
Day 15

**Parameter / Observation**	**Qualitative Urinalysis**				
**Dose (µg/animal)**	**0**	**10**	**25**	**0**	**25**
**Groups**	**G1**	**G2**	**G3**	**G4**	**G5**
**Number examined**	6	6	6	6	6
**Specific Gravity**					
**Mean**	1.016	1.008	1.007	1.008	1.015
**SD**	0.012	0.008	0.003	0.003	0.008
**pH**					
**Mean**	7.58	8.08	7.67	6.67	7.25
**SD**	1.24	1.11	0.82	0.26	1.04
**Color**					
Yellow	6	6	6	6	6
Dark Yellow	0	0	0	0	0
Amber	0	0	0	0	0
Brown	0	0	0	0	0
**Turbidity**					
Clear	6	6	6	6	6
Turbid	0	0	0	0	0
Cloudy	0	0	0	0	0
**Bilirubin**					
Negative	6	6	6	6	6
Positive	0	0	0	0	0
**Blood**					
Negative	6	6	6	6	6
Positive	0	0	0	0	0
**Glucose**					
Negative	6	6	6	6	6
Positive	0	0	0	0	0
**Protein**					
Negative	6	6	6	6	6
Positive	0	0	0	0	0

Note: SD = Standard Deviation.

.

Supplementary Data S3 (Contd.): Qualitative Urinalysis

Sex: Female **Day 15**

Parameter / Observation	Qualitative Urinalysis				
**Dose (µg/animal)**	**0**	**10**	**25**	**0**	**25**
**Groups**	**G1**	**G2**	**G3**	**G4**	**G5**
**Number examined**	6	6	6	6	6
**Specific Gravity**					
**Mean**	1.020	1.008*	1.005*	1.008	1.009
**SD**	0.004	0.006	0.000	0.009	0.005
**pH**					
**Mean**	6.67	8.08*	8.67*	7.33	7.50
**SD**	0.26	0.92	0.52	0.75	0.55
**Color**					
Yellow	6	6	6	6	6
Dark Yellow	0	0	0	0	0
Amber	0	0	0	0	0
Brown	0	0	0	0	0
**Turbidity**					
Clear	6	6	6	6	6
Turbid	0	0	0	0	0
Cloudy	0	0	0	0	0
**Bilirubin**					
Negative	6	6	6	6	6
Positive	0	0	0	0	0
**Blood**					
Negative	6	6	6	6	6
Positive	0	0	0	0	0
**Glucose**					
Negative	6	6	6	6	6
Positive	0	0	0	0	0
**Protein**					
Negative	6	6	6	6	6
Positive	0	0	0	0	0

Note: SD = Standard Deviation.
Key: * = Mean value of group significantly different from Placebo Control group at p<0.05

Supplementary Data S3 (Contd.): Qualitative Urinalysis

Sex: Male **Day 43**

Parameter / Observation	Qualitative Urinalysis	
**Dose (µg/animal)**	**0**	**25**
**Groups**	**G4**	**G5**
**Number examined**	6	6
**Specific Gravity**		
**Mean**	1.005	1.004
**SD**	0.000	0.002
**pH**		
**Mean**	8.500	7.500
**SD**	0.548	1.761
**Color**		
Yellow	6	6
Dark Yellow	0	0
Amber	0	0
Brown	0	0
**Turbidity**		
Clear	6	6
Turbid	0	0
Cloudy	0	0
**Bilirubin**		
Negative	6	6
Positive	0	0
**Blood**		
Negative	6	6
Positive	0	0
**Glucose**		
Negative	6	6
Positive	0	0
**Protein**		
Negative	6	6
Positive	0	0

Supplementary Data S3 (Contd): Qualitative Urinalysis

Sex: Female **Day 43**

Parameter / Observation	Qualitative Urinalysis	
**Dose (µg/animal)**	**0**	**25**
**Groups**	**G4**	**G5**
**Number examined**	6	6
**Specific Gravity**		
**Mean**	1.003	1.004
**SD**	0.003	0.004
**pH**		
**Mean**	7.42	6.75
**SD**	1.11	1.17
**Color**		
Yellow	6	6
Dark Yellow	0	0
Amber	0	0
Brown	0	0
**Turbidity**		
Clear	6	6
Turbid	0	0
Cloudy	0	0
**Bilirubin**		
Negative	6	6
Positive	0	0
**Blood**		
Negative	6	6
Positive	0	0
**Glucose**		
Negative	6	6
Positive	0	0
**Protein**		
Negative	6	6
Positive	0	0
