# Supplementary material for: Development, Pre-Clinical Safety, and Immune Profile of RENOVAC—A Dimer RBD-Based Anti-Coronavirus Subunit Vaccine
Source: Vaccines (Basel). 2024 Dec 17;12(12):1420. doi: 10.3390/vaccines12121420 (PMC11680381; doi:10.3390/vaccines12121420)
Supplement: Supplementary file 1 [file vaccines-12-01420-s001.zip › Supplementary Data S4.pdf]

Supplementary Data S4: Gross Pathology Observations

Tissue/ Findings/ Sex	Males					Females				
**Dose Group**	**G1**	**G2**	**G3**	**G4**	**G5**	**G1**	**G2**	**G3**	**G4**	**G5**
**Dose (µg/animal)**	**0**	**10**	**25**	**0**	**25**	**0**	**10**	**25**	**0**	**25**
**Number Examined**	6	6	6	6	6	6	6	6	6	6
**NAD**	6	6	6	6	6	6	6	6	6	6

Key: NAD =No Abnormality Detected.
