# Supplementary material for: Development, Pre-Clinical Safety, and Immune Profile of RENOVAC—A Dimer RBD-Based Anti-Coronavirus Subunit Vaccine
Source: Vaccines (Basel). 2024 Dec 17;12(12):1420. doi: 10.3390/vaccines12121420 (PMC11680381; doi:10.3390/vaccines12121420)
Supplement: Supplementary file 1 [file vaccines-12-01420-s001.zip › Supplementary Data S5.pdf]

Supplementary Data S5: Absolute Organ Weight (gm)

Sex: Male **Day 15**

Mean/SD/N	Liver	Spleen	Heart	Thymus	Kidneys	Adrenals	Testes	Brain
**G1 Placebo Control**								**Dose: 0 µg/animal**
**Mean**	10.02	0.73	0.82	0.27	1.95	0.029	2.73	1.76
**SD**	2.33	0.15	0.12	0.09	0.38	0.010	0.15	0.16
**N**	6	6	6	6	6	6	6	6
**G2 Low Dose**								**Dose: 10 µg/animal**
**Mean**	9.85	0.73	0.85	0.23	1.85	0.023	2.64	1.66
**SD**	0.65	0.22	0.06	0.07	0.11	0.006	0.17	0.06
**N**	6	6	6	6	6	6	6	6
**G3 High Dose**								**Dose: 25 µg/animal**
**Mean**	10.07	0.90	0.81	0.26	1.91	0.032	2.63	1.73
**SD**	1.00	0.26	0.07	0.10	0.19	0.009	0.22	0.12
**N**	6	6	6	6	6	6	6	6

Day 43

Mean/SD/N	Liver	Spleen	Heart	Thymus	Kidneys	Adrenals	Testes	Brain
**G4 Placebo Control-R**								**Dose: 0 µg/animal**
**Mean**	13.55	0.84	1.07	0.31	2.93	0.049	3.11	2.09
**SD**	2.40	0.18	0.10	0.11	0.29	0.010	0.22	0.34
**N**	6	6	6	6	6	6	6	6
**G5 High Dose-R**								**Dose: 25 µg/animal**
**Mean**	11.84	0.78	1.04	0.34	2.70	0.037	2.98	1.98
**SD**	1.46	0.25	0.10	0.09	0.50	0.014	0.21	0.26
**N**	6	6	6	6	6	6	6	6

Note: N = number of animals; SD = Standard Deviation.

Supplementary Data S5: (Contd.): Absolute Organ Weight (gm)

Sex: Female **Day 15**

Mean/SD/N	Liver	Spleen	Heart	Thymus	Kidneys	Adrenals	Testes	Brain
**G1 Placebo Control**								**Dose: 0 µg/animal**
**Mean**	7.64	0.63	0.65	0.28	1.47	0.045	0.053	1.67
**SD**	1.02	0.21	0.03	0.10	0.16	0.007	0.004	0.06
**N**	6	6	6	6	6	6	6	6
**G2 Low Dose**								**Dose: 10 µg/animal**
**Mean**	7.57	0.61	0.73*	0.25	1.50	0.052	0.061	1.75
**SD**	0.93	0.14	0.05	0.03	0.10	0.012	0.018	0.06
**N**	6	6	6	6	6	6	6	6
**G3 High Dose**								**Dose: 25 µg/animal**
**Mean**	7.79	0.58	0.72*	0.31	1.50	0.041	0.057	1.74
**SD**	0.37	0.20	0.04	0.09	0.08	0.015	0.009	0.08
**N**	6	6	6	6	6	6	6	6

Day 43

Mean/SD/N	Liver	Spleen	Heart	Thymus	Kidneys	Adrenals	Testes	Brain
**G4 Placebo Control-R**								**Dose: 0 µg/animal**
**Mean**	9.26	0.67	0.82	0.29	2.07	0.070	0.076	1.95
**SD**	1.53	0.18	0.05	0.05	0.15	0.010	0.021	0.17
**N**	6	6	6	6	6	6	6	6
**G5 High Dose-R**								**Dose: 25 µg/animal**
**Mean**	9.73	0.70	0.85	0.39*	2.39	0.074	0.070	2.04
**SD**	1.31	0.13	0.07	0.06	0.37	0.025	0.014	0.31
**N**	6	6	6	6	6	6	6	6

Note: N = number of animals; SD = Standard Deviation.
Key: * = Mean value of group significantly different from Placebo Control group at $p<0.05$.
