# Supplementary material for: Development, Pre-Clinical Safety, and Immune Profile of RENOVAC—A Dimer RBD-Based Anti-Coronavirus Subunit Vaccine
Source: Vaccines (Basel). 2024 Dec 17;12(12):1420. doi: 10.3390/vaccines12121420 (PMC11680381; doi:10.3390/vaccines12121420)
Supplement: Supplementary file 1 [file vaccines-12-01420-s001.zip › Supplementary Data S6_Histopathology-Images-Female.pdf]

Study Number: PRADO/TOX-504- Female

Control (G1)	High dose (G3)
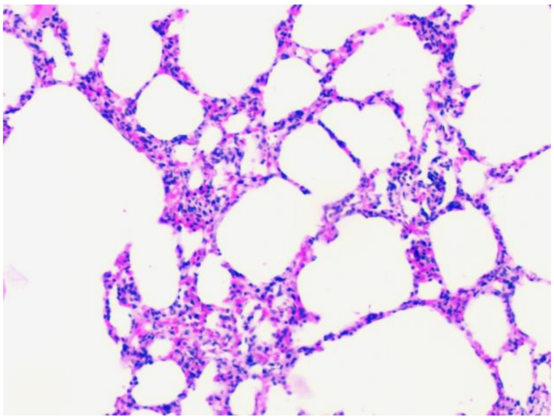	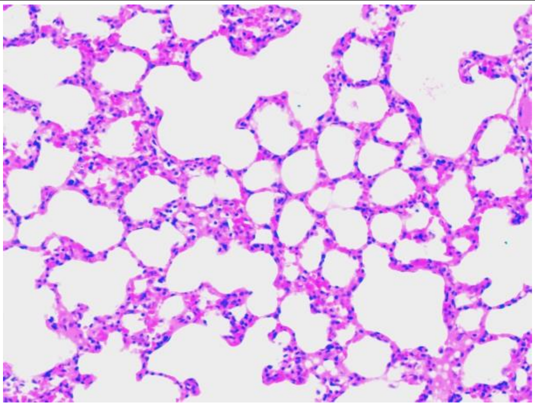
Lungs: No abnormality Detected, H & E, 10X	Lungs: No abnormality Detected, H & E, 10X
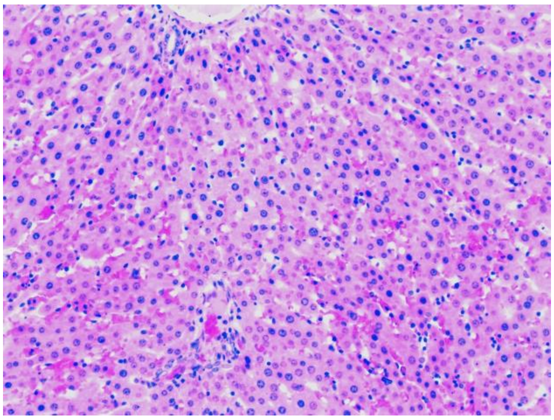	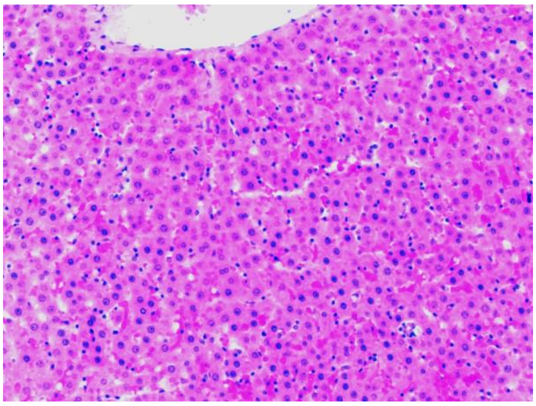
Liver: No abnormality Detected, H & E, 10X	Liver: No abnormality Detected, H & E, 10X
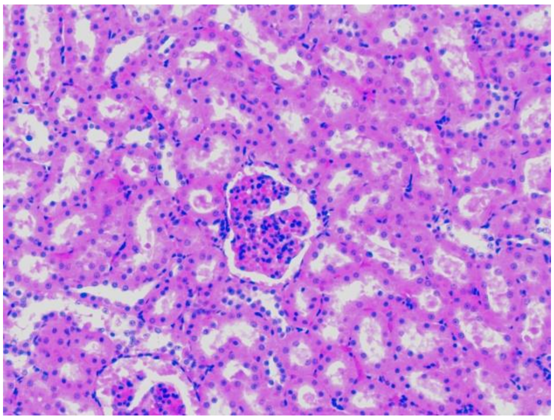	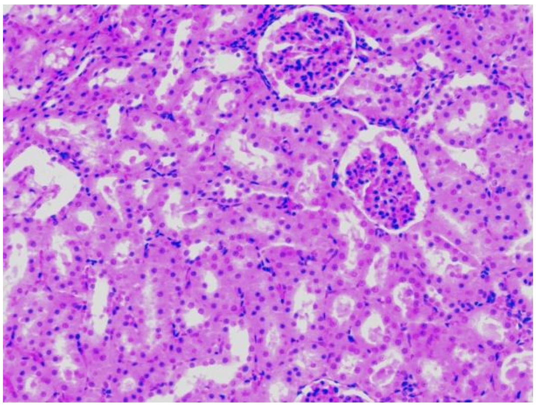
Kidney: No abnormality Detected, H & E, 10X	Kidney: No abnormality Detected, H & E, 10X

**Control (G1)**	**High dose (G3)**
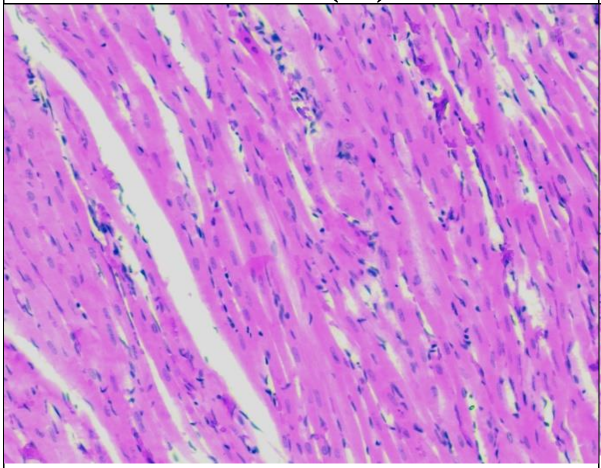	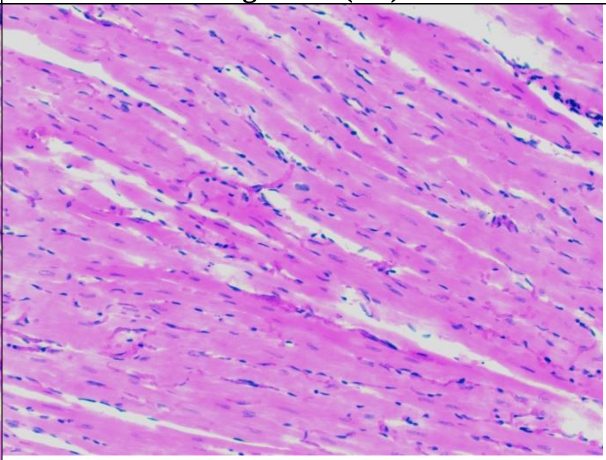
Heart: No abnormality Detected, H & E, 10X	Heart: No abnormality Detected, H & E, 10X
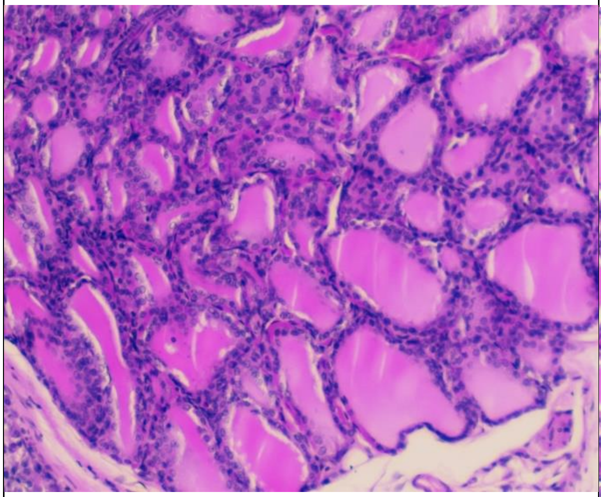	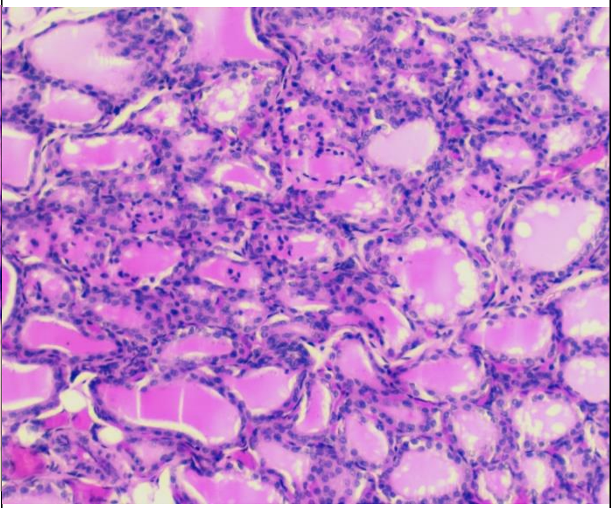
Thyroid: No abnormality Detected, H & E, 10X	Thyroid: No abnormality Detected, H & E, 10X
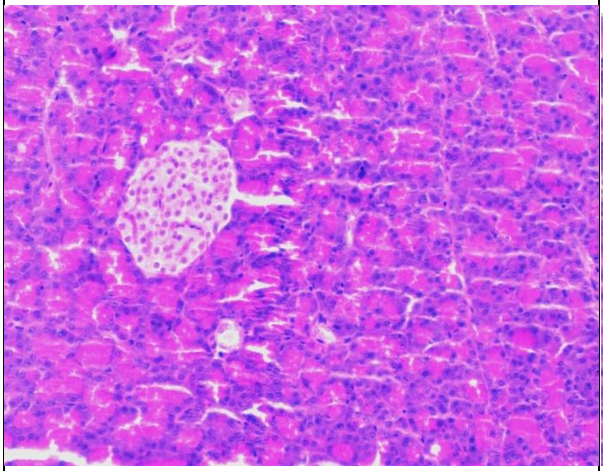	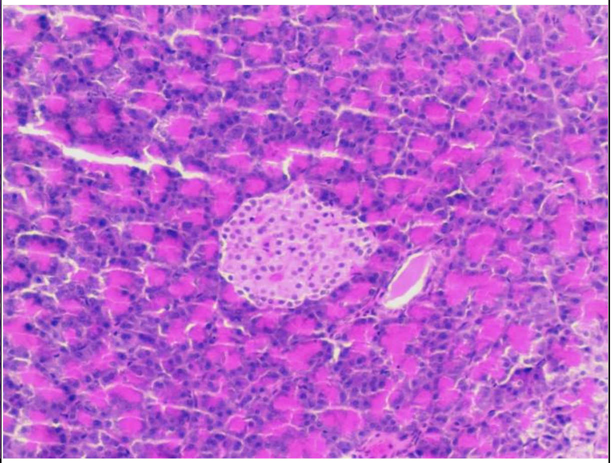
Pancreas: No abnormality Detected, H & E, 10X	Pancreas: No abnormality Detected, H & E, 10X

**Control (G1)**	**High dose (G3)**
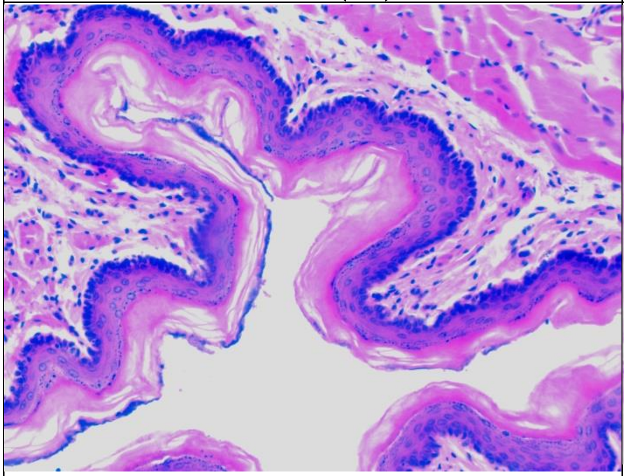	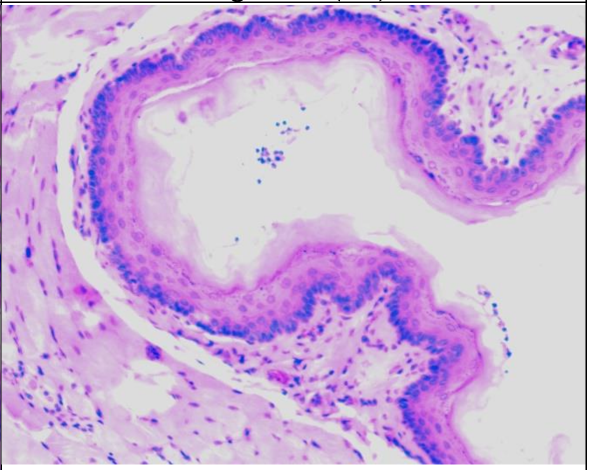
Oesophagus: No abnormality Detected, H & E, 10X	Oesophagus: No abnormality Detected, H & E, 10X
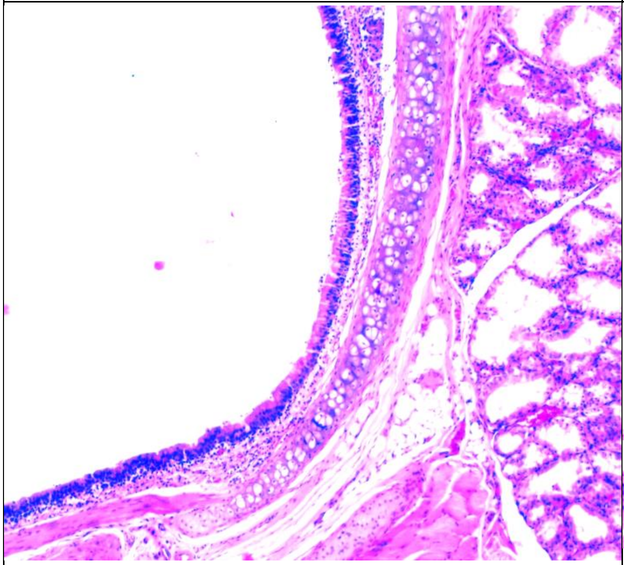	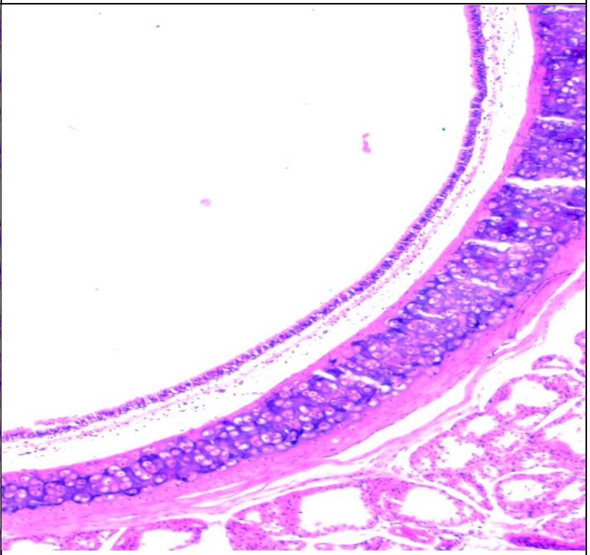
Trachea: No abnormality Detected, H & E, 10X	Trachea: No abnormality Detected, H & E, 10X
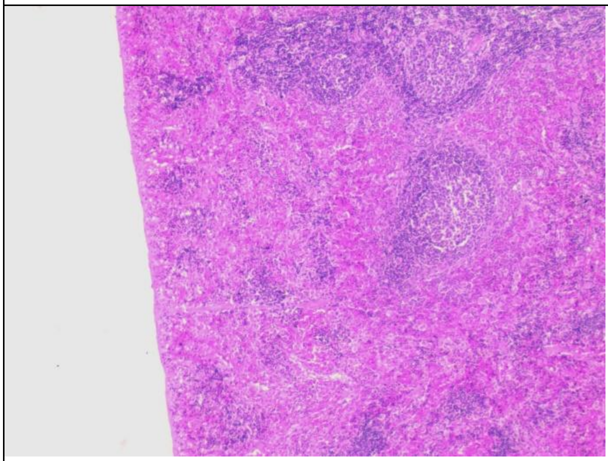	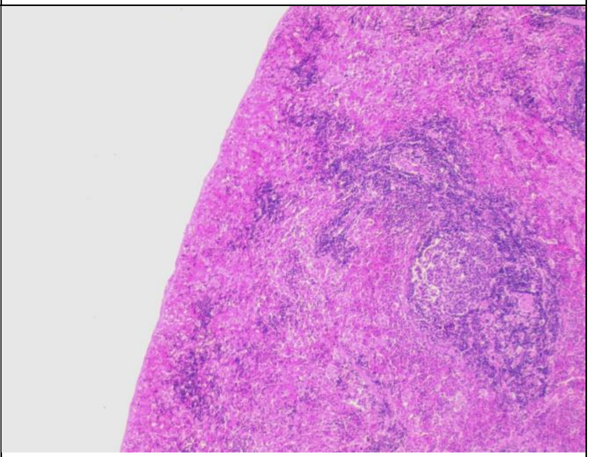
Spleen: No abnormality Detected, H & E, 4X	Spleen: No abnormality Detected, H & E, 4X

**Control (G1)**	**High dose (G3)**
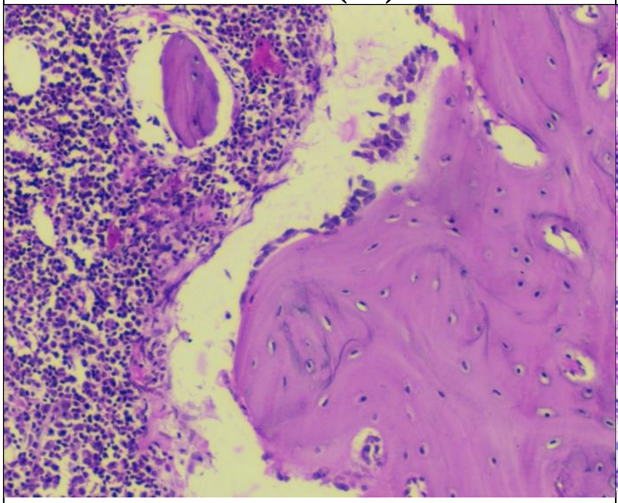	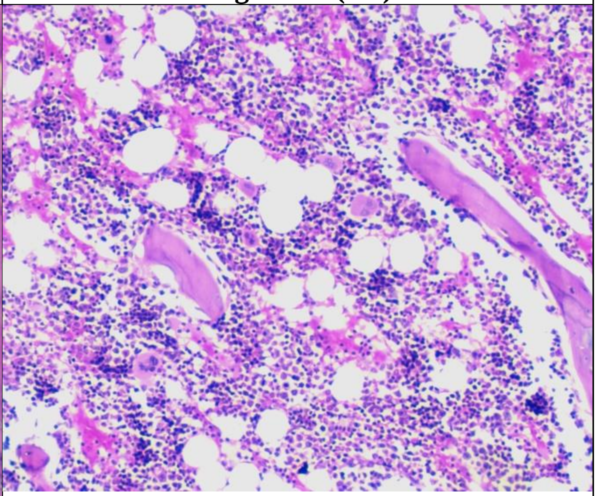
Bone (Bone marrow): No abnormality Detected, H & E, 10X	Bone (Bone marrow): No abnormality Detected, H & E, 10X
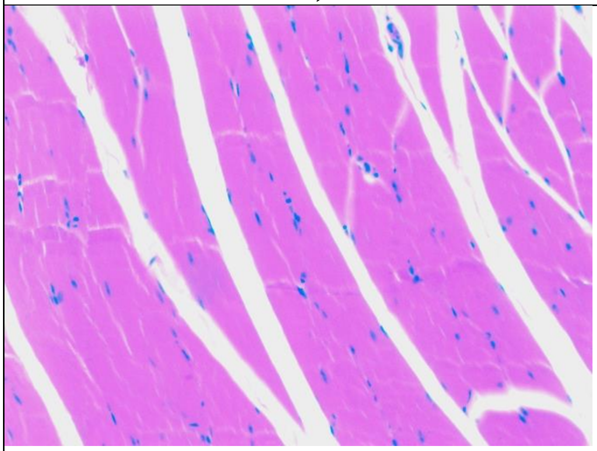	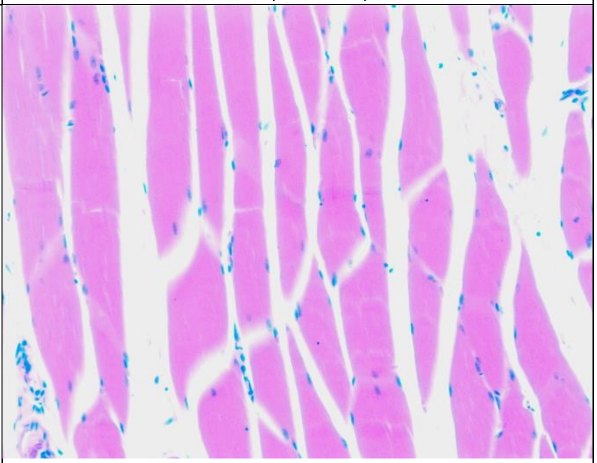
Skeletal Muscle: No abnormality Detected, H & E, 10X	Skeletal Muscle: No abnormality Detected, H & E, 10X
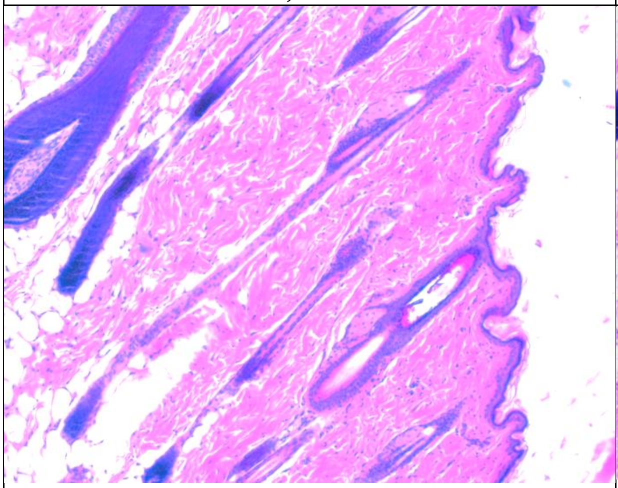	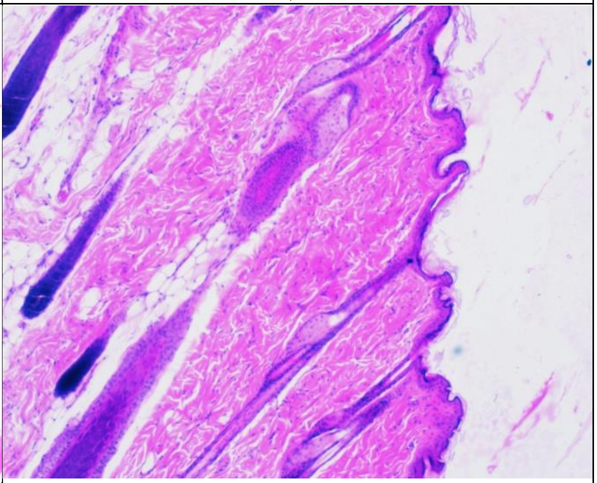
Skin: No abnormality Detected, H & E, 4X	Skin: No abnormality Detected, H & E, 4X

**Control (G1)**	**High dose (G3)**
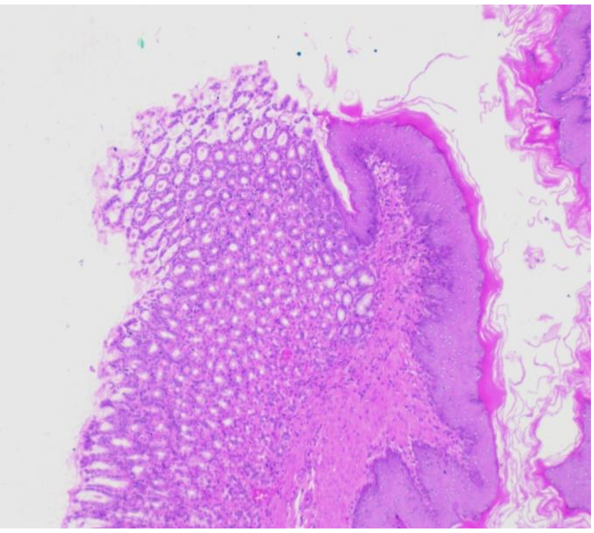	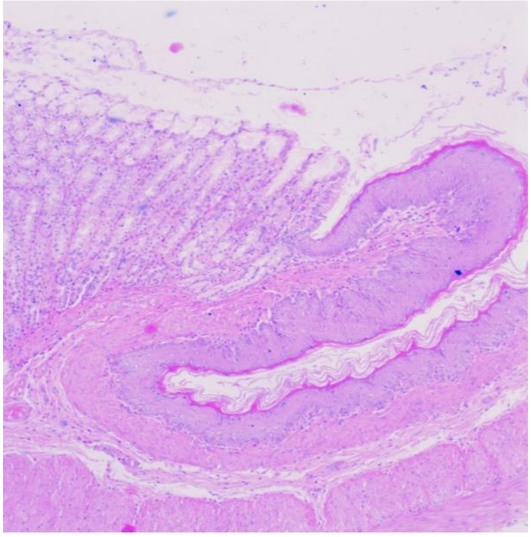
Stomach: No abnormality Detected, H & E, 4X	Stomach: No abnormality Detected, H & E, 4X
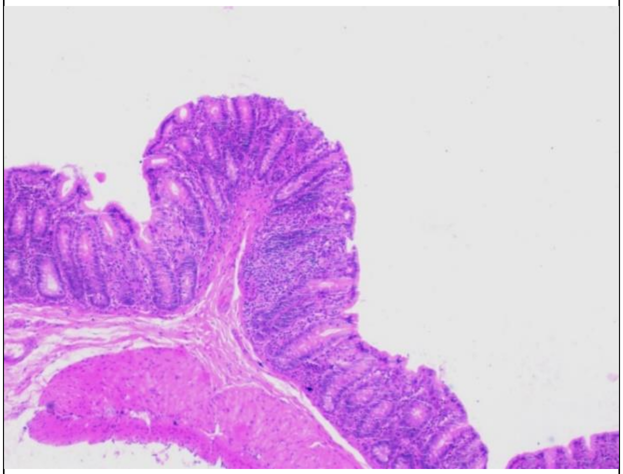	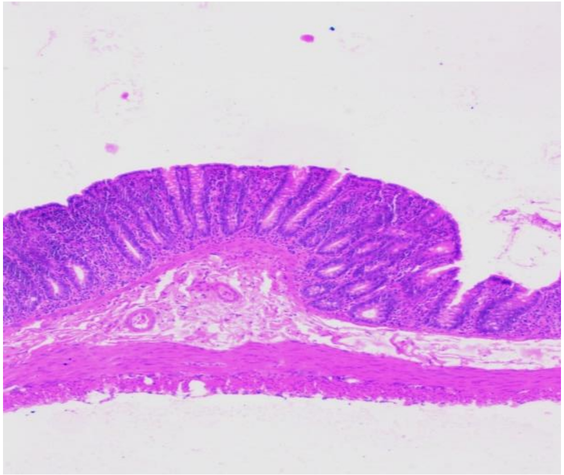
Large Intestine: No abnormality Detected, H & E, 4X	Large Intestine: No abnormality Detected, H & E, 4X
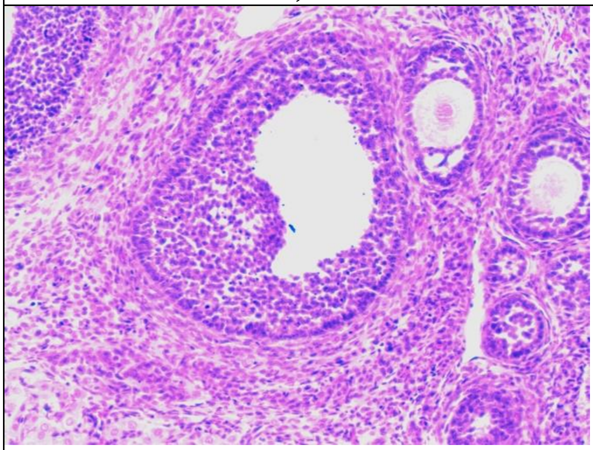	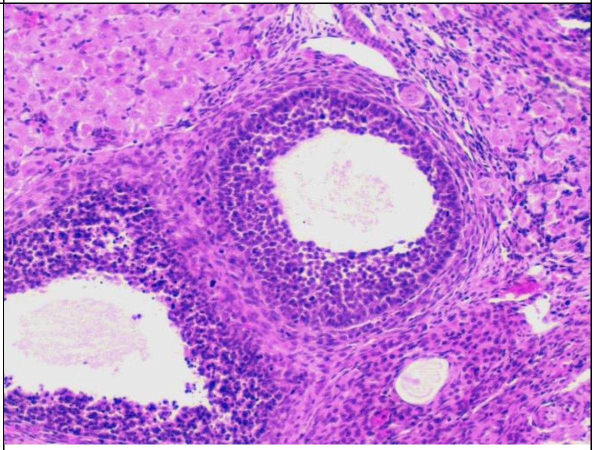
Ovaries: No abnormality Detected, H & E, 10X	Ovaries: No abnormality Detected, H & E, 10X

**Control (G1)**	**High dose (G3)**
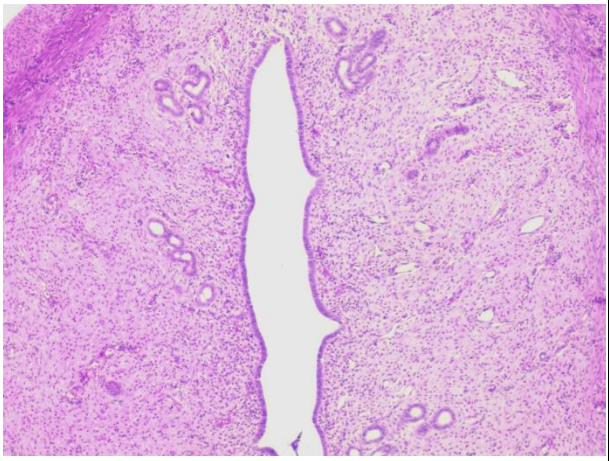	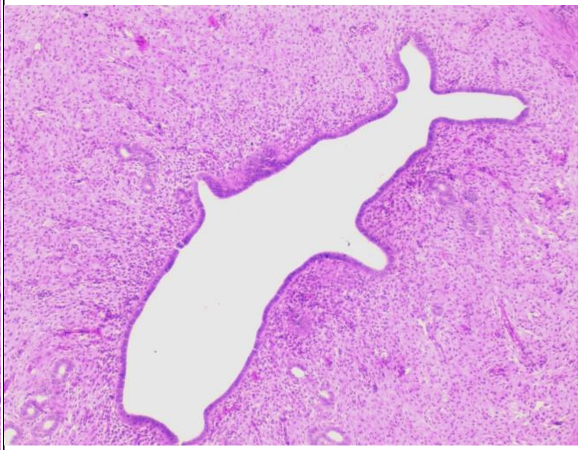
Uterus: No abnormality Detected, H & E, 10X	Uterus: No abnormality Detected, H & E, 10X
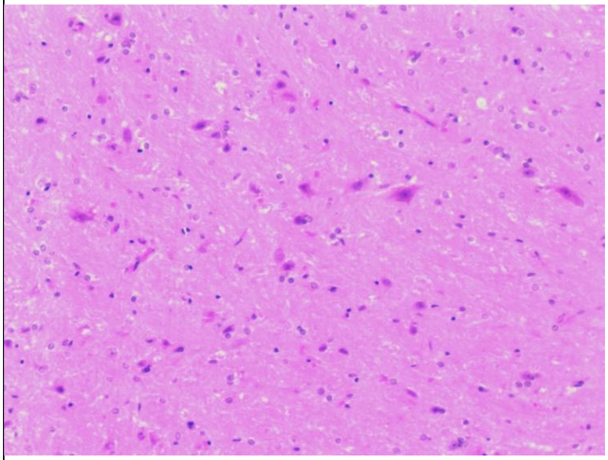	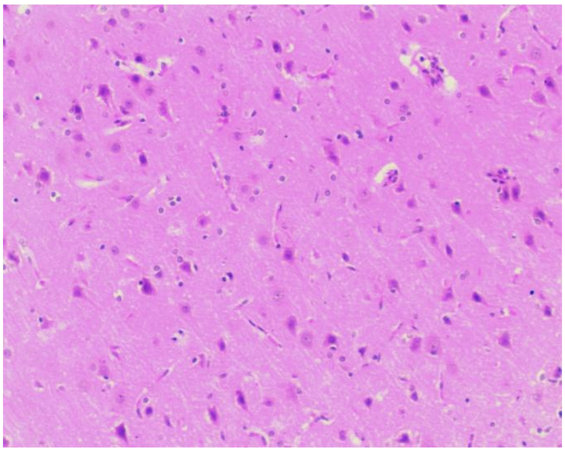
Brain: No abnormality Detected, H & E, 10X	Brain: No abnormality Detected, H & E, 10X
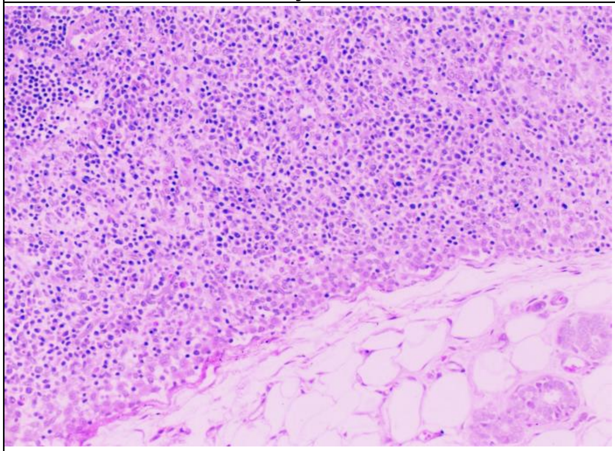	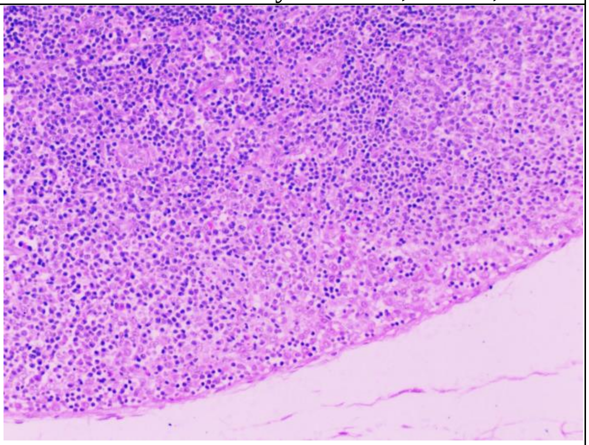
Lymphnode: No abnormality Detected, H & E, 10X	Lymphnode: No abnormality Detected, H & E, 10X

<table>
<tr><th>Control (G1)</th><th>High dose (G3)</th></tr>
<tr><td>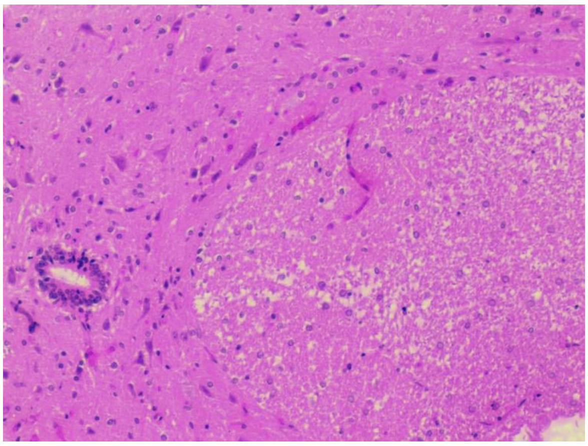</td><td>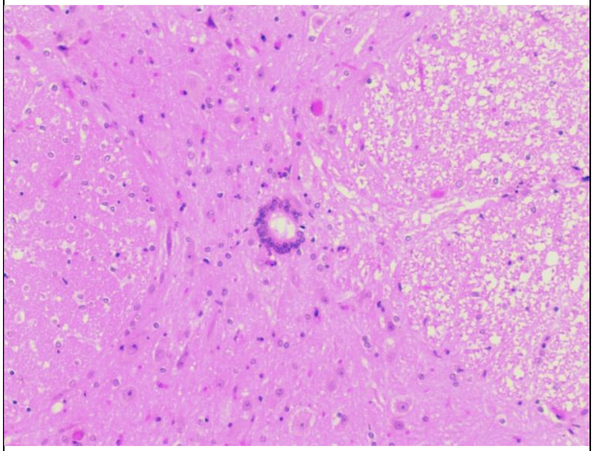</td></tr>
<tr><td>Spinal Cord: No abnormality Detected, H & E, 10X</td><td>Spinal Cord: No abnormality Detected, H & E, 10X</td></tr>
<tr><td>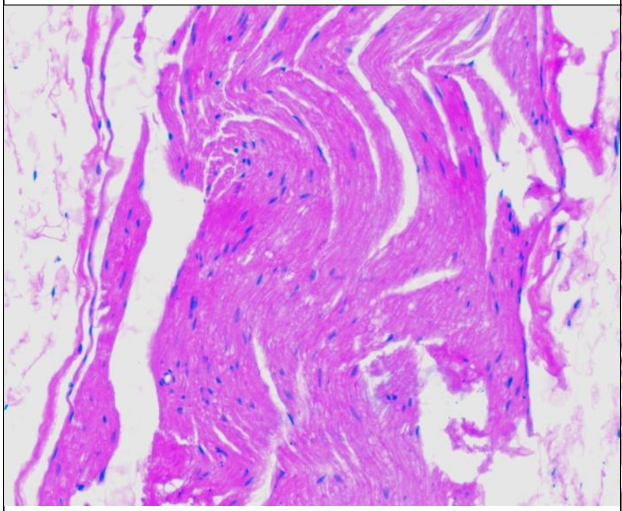</td><td>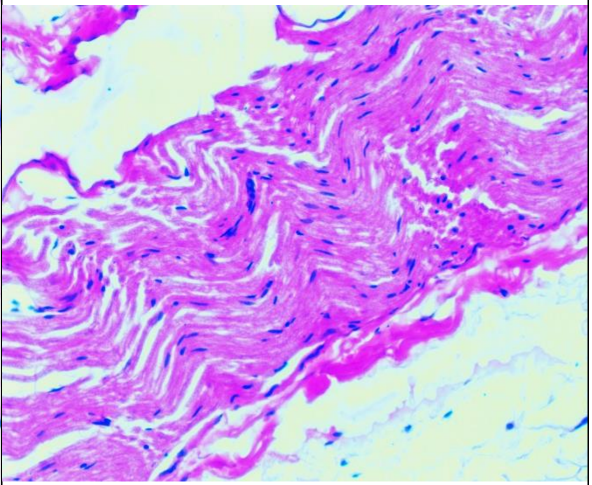</td></tr>
<tr><td>Sciatic Nerve: No abnormality Detected, H & E, 10X</td><td>Sciatic Nerve: No abnormality Detected, H & E, 10X</td></tr>
<tr><td>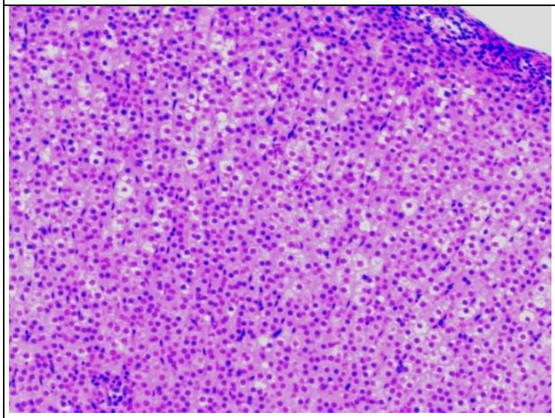</td><td>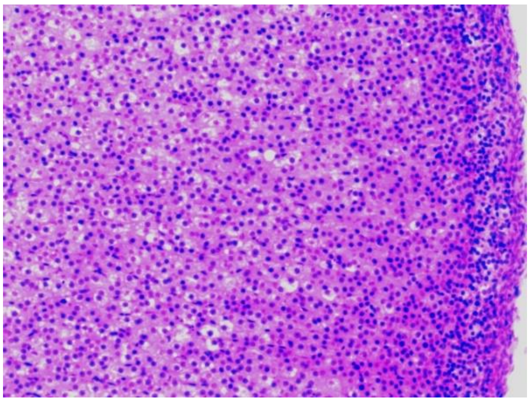</td></tr>
<tr><td>Adrenals: No abnormality Detected, H & E, 10X</td><td>Adrenals: No abnormality Detected, H & E, 10X</td></tr>
</table>

**Control (G1)**	**High dose (G3)**
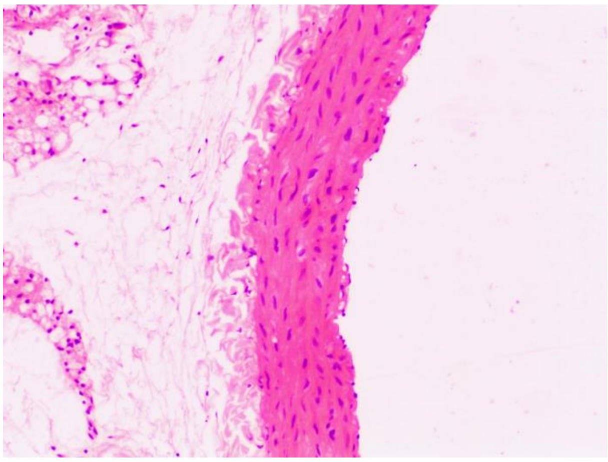	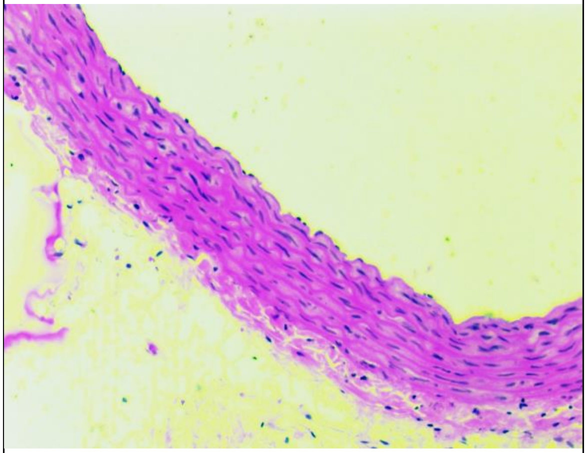
Aorta: No abnormality Detected, H & E, 10X	Aorta: No abnormality Detected, H & E, 10X
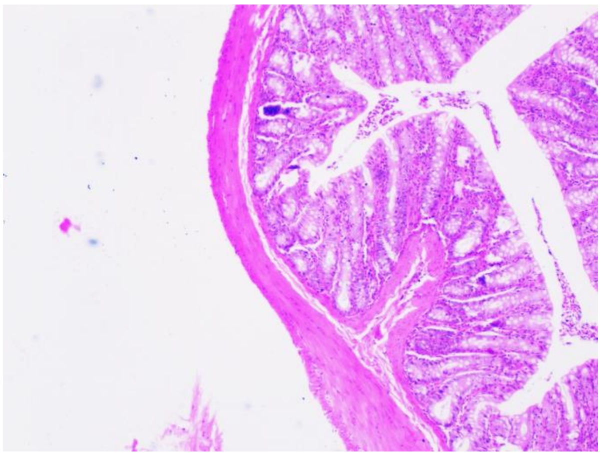	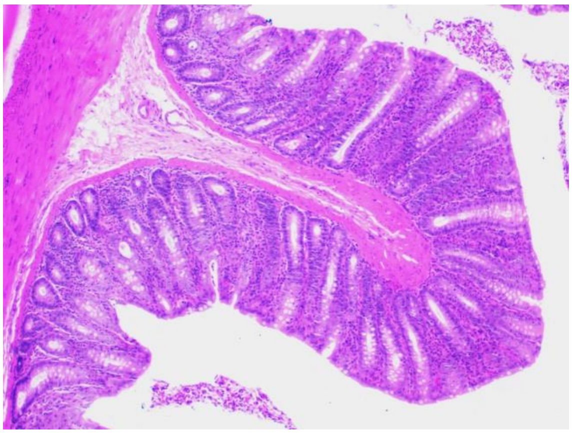
Small Intestine: No abnormality Detected, H & E, 4X	Small Intestine: No abnormality Detected, H & E, 4X
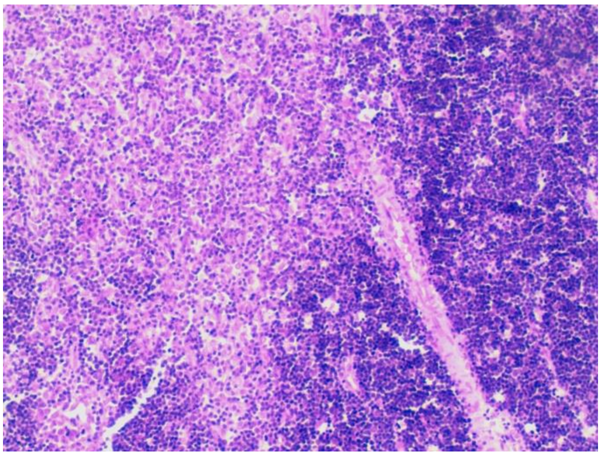	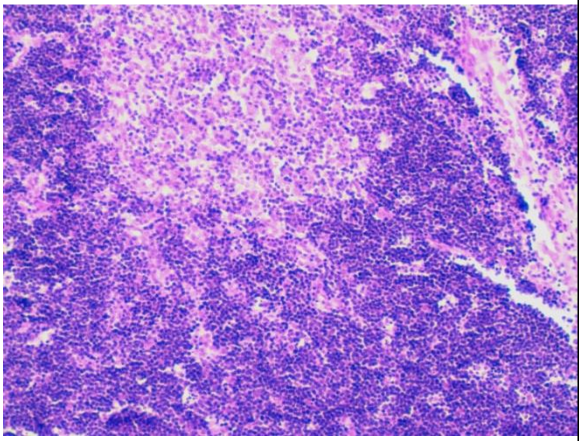
Thymus: No abnormality Detected, H & E, 10X	Thymus: No abnormality Detected, H & E, 10X
